# Comparative genomic analysis of *Mycobacterium intracellulare*: implications for clinical taxonomic classification in pulmonary *Mycobacterium avium-intracellulare* complex disease

**DOI:** 10.1186/s12866-021-02163-9

**Published:** 2021-04-06

**Authors:** Yoshitaka Tateishi, Yuriko Ozeki, Akihito Nishiyama, Mari Miki, Ryoji Maekura, Yukari Fukushima, Chie Nakajima, Yasuhiko Suzuki, Sohkichi Matsumoto

**Affiliations:** 1 Department of Bacteriology, Graduate School of Medical and Dental Sciences, Niigata University, Niigata, Japan; 2 Department of Respiratory Medicine, National Hospital Organization Osaka Toneyama Medical Center, Toyonaka, Osaka, Japan; 3 Graduate School of Health Care Sciences, Jikei Institute, Osaka, Japan; 4 Division of Bioresources, Hokkaido University Research Center for Zoonosis Control, Sapporo, Japan; 5 International Collaboration Unit, Research Center for Zoonosis Control, Hokkaido University, Sapporo, Japan; 6 Laboratory of Tuberculosis, Institute of Tropical Disease, Universitas Airlangga, Kampus C Jl. Mulyorejo, Surabaya, Indonesia

**Keywords:** *Mycobacterium intracellulare*, *Mycobacterium paraintracellulare*, *Mycobacterium indicus pranii*, Comparative genomics, Mammalian cell entry genes

## Abstract

**Background:**

*Mycobacterium intracellulare* is a representative etiological agent of emerging pulmonary *M. avium-intracellulare* complex disease in the industrialized countries worldwide. The recent genome sequencing of clinical strains isolated from pulmonary *M. avium-intracellulare* complex disease has provided insight into the genomic characteristics of pathogenic mycobacteria, especially for *M. avium*; however, the genomic characteristics of *M. intracellulare* remain to be elucidated.

**Results:**

In this study, we performed comparative genomic analysis of 55 *M. intracellulare* and related strains such as *M. paraintracellulare* (MP), *M. indicus pranii* (MIP) and *M. yonogonense*. Based on the average nucleotide identity, the clinical *M. intracellulare* strains were phylogenetically grouped in two clusters: (1) the typical *M. intracellulare* (TMI) group, including ATCC13950 and virulent M.i.27 and M.i.198 that we previously reported, and (2) the MP-MIP group. The alignment of the genomic regions was mostly preserved between groups. Plasmids were identified between groups and subgroups, including a plasmid common among some strains of the M.i.27 subgroup. Several genomic regions including those encoding factors involved in lipid metabolism (e.g., *fadE3*, *fadE33*), transporters (e.g., *mce3*), and type VII secretion system (genes of ESX-2 system) were shown to be hypermutated in the clinical strains. *M. intracellulare* was shown to be pan-genomic at the species and subspecies levels. The *mce* genes were specific to particular subspecies, suggesting that these genes may be helpful in discriminating virulence phenotypes between subspecies.

**Conclusions:**

Our data suggest that genomic diversity among *M. intracellulare*, *M. paraintracellulare*, *M. indicus pranii* and *M. yonogonense* remains at the subspecies or genovar levels and does not reach the species level. Genetic components such as *mce* genes revealed by the comparative genomic analysis could be the novel focus for further insight into the mechanism of human pathogenesis for *M. intracellulare* and related strains.

**Supplementary Information:**

The online version contains supplementary material available at 10.1186/s12866-021-02163-9.

## Background

Mycobacteria have successfully adapted to the human host environment as represented by the worldwide prevalence of tuberculosis and nontuberculous mycobacteriosis [1]. *Mycobacterium avium* and *Mycobacterium intracellulare* (MAC) are major causative agents of pulmonary nontuberculous mycobacterial disease in Europe, the US, and Asia, including Japan [2–5]. The identification of genetic differences between species and strains provides basic information for investigating the cause of differential epidemiology, virulence, and pathogenesis.

Recent advances in genome sequencing technology have enabled the comparison of clinically-isolated strains at the whole genome level and this has revealed that some mycobacterial species have open pan-genomes [6–9]. In *M. avium*, the genomic diversity is classified into several subspecies, including subspecies *avium*, *hominissuis*, *paratuberculosis*, and *silvaticum* [10]. *M. avium* subsp. *avium* causes avian tuberculosis in birds and other domestic animals such as pigs and cattle [11]. *M. avium* subsp. *hominissuis* causes pulmonary infections, cervical lymphadenitis, soft tissue infections and disseminated infections in humans and porcine [11]. *M. avium* subsp. *paratuberculosis* causes Johne’s disease in ruminants and non-ruminant domestic animals (pigs, dogs, horses, cats, etc.), free ranging animals (rabbits, bears, rats, etc.) and non-human primates [12]. *M. avium* subsp. *silvaticum* causes tuberculosis-like disease in wood pigeons, cranes and deer [12]. Strains belonging to some clusters have been suggested to possess potential genetic factors that account for their clinical pathogenesis [9].

In *M. intracellulare*, the comparative genomic approach has not been reported for typing strains to date; nevertheless, new relevant species and subspecies have been proposed by multi-locus sequencing typing of a single strain, i.e., *M. paraintracellulare* (MP) [13], *M. yongonense* [14] and *M. indicus pranii* (MIP) [15, 16]. However, the grouping of *M. intracellulare* and its related strains is confusing in the NCBI genome database. As for February 2021, *M. yongonense* and MIP are registered as an independent subspecies of *M. intracellulare*, i.e. *M. intracellulare* subsp. *yongonense* and *M. intracellulare* subsp. *intracellulare*. And curiously, only one MIP strain, MTCC9506 belongs to *M. intracellulare* subsp. *intracellulare* on the NCBI genome database, in spite of a dozen of *M. intracellulare* strains (including the type strain ATCC13950) registered in a category of *M. intracellulare* species without designation of their subspecies. In addition, MP, whose reference strain was originally reported as *M. intracellulare* MOTT64 [17], is registered as an independent species in the NCBI genome database. The actual grouping of these strains, along with a large number of clinically-isolated strains, remains to be elucidated. According to some reports from South Korea, about 4 and 3% of strains diagnosed as *M. intracellulare* by a commercial hybridization assay of *rpoB* gene have been identified as MIP and *M. yongonense* by sequence-based typing analyses, respectively [18]. Although typing of strains based on the sequence of housekeeping genes is a quick and effective method for classifying clinical strains, the method of species verification at the single strain level needs scientific verification by comparative genomic analysis, as proposed by the comprehensive taxonomic analysis of nontuberculous mycobacteria [19, 20].

We have reported the virulence of clinical *M. intracellulare* strains (strains M.i.198 and M.i.27) in comparison with clinical *M. avium* strains [21]. We also identified a 50-kb region of a prophage in the hypervirulent strain M.i.198 [22]. These data prompted us to investigate in more depth the genomic landscape at the multiple strain level. To understand the genomic similarity and diversity between clinically-isolated *M. intracellulare* strains, and previously-reported *M. intracellulare* and related species and subspecies, we performed comparative genomic analysis of *M. intracellulare* for 55 strains including 31 clinical strains (including M.i.198 and M.i.27) isolated from cases of pulmonary *M. intracellulare* disease. We clarified the classification of clinical *M. intracellulare* strains into two major groups: the typical *M. intracellulare* (TMI) group and the *M. paraintracellulare - M. indicus pranii* (MP-MIP) group. The genomic difference was not significant enough that species differentiation between the strains enrolled in this study was possible by means of the genomic alignment and the nucleotide identity. Therefore, we propose new insights into the clinical taxonomic classification of *M. intracellulare* and related strains.

## Results

### Identification of the species of clinical strains and general genomic features

The clinical strains were assigned to species based on the best match strain among the NCBI reference strains (Table 1). In total, 17, 3, 5, and 6 strains were assigned to *M. intracellulare* ATCC13950, *M. intracellulare* MOTT-02, MP, and MIP, respectively. The percentage of mapped regions was high, ranging from 85 to 100%. These data suggested that MP and MIP comprised approximately one-third of clinical strains diagnosed as *M. intracellulare*. No clinical strains were identified as *M. yongonense*.

**Table 1:** Genomic features of the *M. intracellulare* and related strains enrolled in this study

	Strain name (GenBank Accession number)	Best match strain on NCBI references for clinical strains, or category in NCBI database for NCBI-registered strains	Best match, % mapped
Clinical strains	M.i.198	*M. intracellulare* MOTT-02	98
	M.i.27	*M. intracellulare* ATCC 13950	87
	M001	*M. paraintracellulare*	90
	M002	*M. intracellulare* ATCC 13950	95
	M003	*M. indicus pranii*	95
	M004	*M. intracellulare* MOTT-02	98
	M005	*M. intracellulare* ATCC 13950	94
	M006	*M. intracellulare* ATCC 13950	92
	M007	*M. intracellulare* ATCC 13950	94
	M008	*M. intracellulare* ATCC 13950	89
	M009	*M. intracellulare* ATCC 13950	90
	M010	*M. intracellulare* ATCC 13950	96
	M011	*M. indicus pranii*	97
	M012	*M. indicus pranii*	93
	M013	*M. intracellulare* ATCC 13950	98
	M014	*M. intracellulare* ATCC 13950	100
	M015	*M. indicus pranii*	95
	M016	*M. intracellulare* ATCC 13950	95
	M017	*M. paraintracellulare*	92
	M018	*M. intracellulare* ATCC 13950	85
	M019	*M. paraintracellulare*	92
	M020	*M. paraintracellulare*	92
	M021	*M. paraintracellulare*	92
	M022	*M. intracellulare* MOTT-02	100
	M023	*M. intracellulare* ATCC 13950	90
	M024	*M. intracellulare* ATCC 13950	94
	M025	*M. intracellulare* ATCC 13950	92
	M026	*M. intracellulare* ATCC 13950	88
	M027	*M. indicus pranii*	95
	M028	*M. intracellulare* ATCC 13950	90
	M029	*M. indicus pranii*	93
NCBI-registered strains	ATCC13950(NC_016946.1)	*M. intracellulare*	
	1956(JAOG01.1)	*M. intracellulare*	
	CSURP8077(CAAHFM01.1)	*M. intracellulare*	
	FLAC0133(NZ_CP023146.1)	*M. intracellulare*	
	FLAC0181(NZ_CP023149)	*M. intracellulare*	
	2285(JAOD01.1)	*M. intracellulare*	
	MIN_061107_1834(JAOM01.1)	*M. intracellulare*	
	MIN052511_1280(JAON01.1)	*M. intracellulare*	
	852002-53206_SCH5915646(LZIO01.1)	*M. intracellulare*	
	E3191(LZJO01.1)	*M. intracellulare*	
	E2190(LZJT01.1)	*M. intracellulare*	
	MOTT-02(NC_016947.1)	*M. intracellulare*	
	FLAC0204(NSFC01.1)	*M. intracellulare*	
	FLAC0162(NSFE01.1)	*M. intracellulare*	
	MTCC9506(NC_018612)	*M. indicus pranii*	
	JCM30622(NZ_AP022597)	*M. paraintracellulare*	
	MOTT64(NC_016948)	*M. paraintracellulare*	
	KCTC29084(NCXN01.1)	*M. paraintracellulare*	
	05–1390(NC_021715)	*M. yongonense*	
	Asan36527(NZ_CP015965)	*M. yongonense*	
	Asan36912(CP015964)	*M. yongonense*	
	1099801.4(MBDX01.1)	*M. yongonense*	
	E3170(MBDZ01.1)	*M. yongonense*	
	RT955(PSQD01.1)	*M. yongonense*	

### Phylogenetic analysis based on whole genome comparisons

**Fig. 1:**
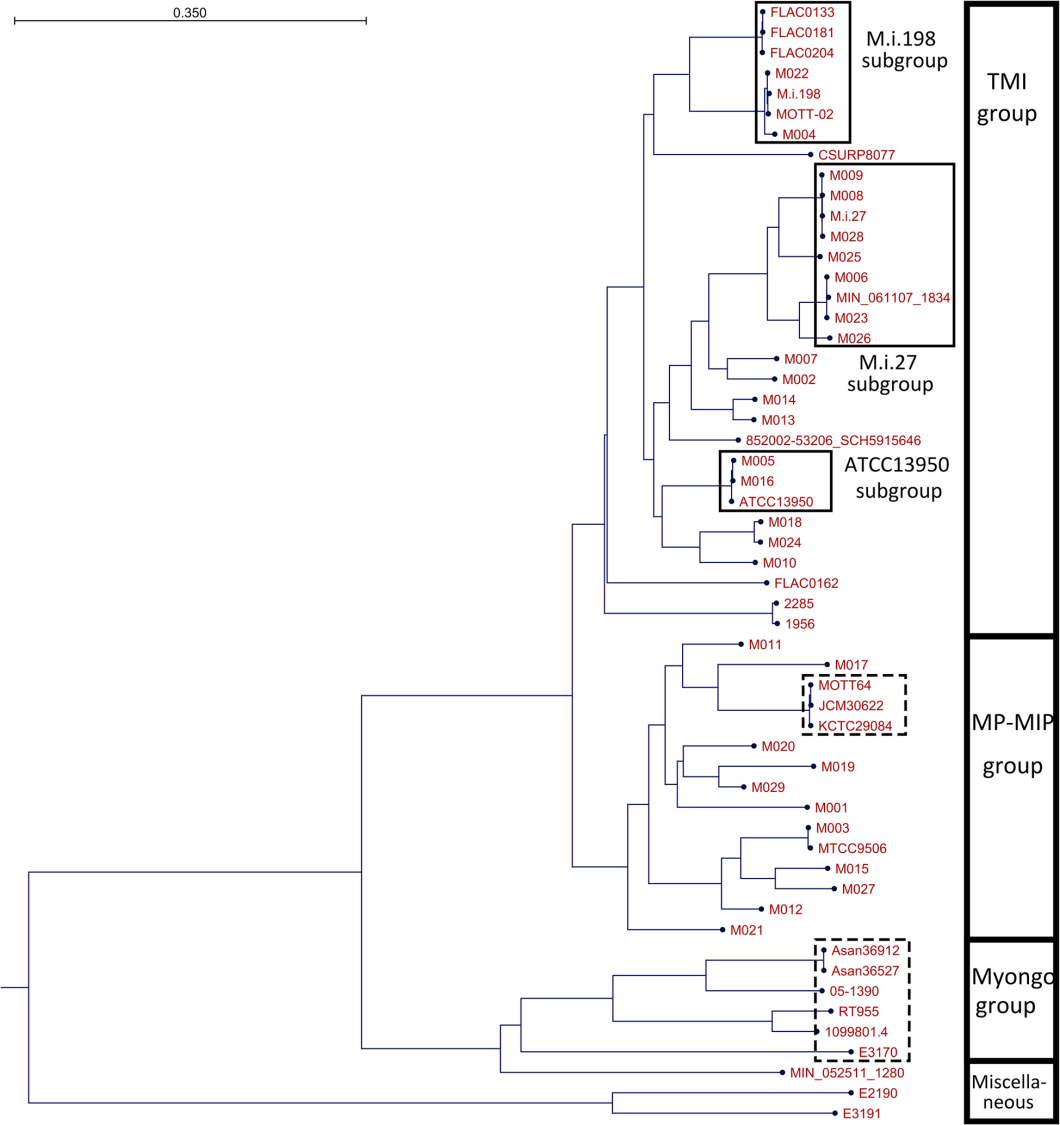
Phylogenetic tree of clinical *M. intracellulare* strains generated based on average nucleotide identity (ANI) using the Neighbour-Joining method. Grouping is represented in the right column (TMI: typical *M. intracellulare*, MP-MIP: *M. paraintracellulare*-*M. indicus pranii,* Myongo: *M. yongonense*). Strains in broken squares indicate those of *M. paraintracellulare* and *M. yongonense* that have previously been registered in the NCBI database

The strains enrolled in this study were grouped into two major groups: the typical *M. intracellulare* (TMI) group and the *M. paraintracellulare-M. indicus pranii* (MP- MIP) group (Fig. 1, Fig. S1). In the TMI group, there were three major subgroups including ATCC13950, M.i.27 and M.i.198 as representative strains, and several minor subgroups including M007, M002, M014, M013, M018, M024 and M010. The subgroups of ATCC13950 included M005 and M016. The subgroup of M.i.27 included M009, M008, M028, M025, M006, M023 and M026. The subgroup of M.i.198 included 4 previously-registered strains including MOTT-02, FLAC01363, FLAC0181 and FLAC0204 and our clinical strains including M022 and M004.

Some strains classified in the MP-MIP group, such as M017 (MP designated by the best match strain in the NCBI database) and M003 (MIP designated by the best match strain in the NCBI database), were shown to be phylogenetically closely related to previously reported *M. paraintracellulare* strains (MOTT64, KCTC29084, and JCM30622) and *M. indicus pranii* MTCC9506, respectively. However, the majority of strains belonging to the MP-MIP group were classified into several subgroups that were closely related to MP and MIP species. With the exception of some parts of the genome registered in the NCBI database showing sequence differences within 3 bp (05–1390, E3191, E2190, FLAC0204, FLAC0181) or cases for which the 16S rRNA sequence could not be identified due to the separation of the contigs (MIN_061107_1834, MIN052511_1280), the sequence of the 16S rRNA was the same between strains in this study (Fig. S2). Considering that the average nucleotide identity and alignment percentage of the strains enrolled in this study were greater than 96% and more than 70% (Fig. S3), *M. paraintracellulare* (including *M. indicus pranii* as a strain of *M. paraintracellulare*) and *M. yongonense* should be classified below the rank of the species of *M. intracellulare* rather than a new species. Consistent to the previous reports [19, 20], *M. avium* strains were phylogenetically placed much far from the *M. intracellulare* and related strains enrolled in this study (Fig. S4).

### Detection of plasmids in clinical strains

**Fig. 2:**
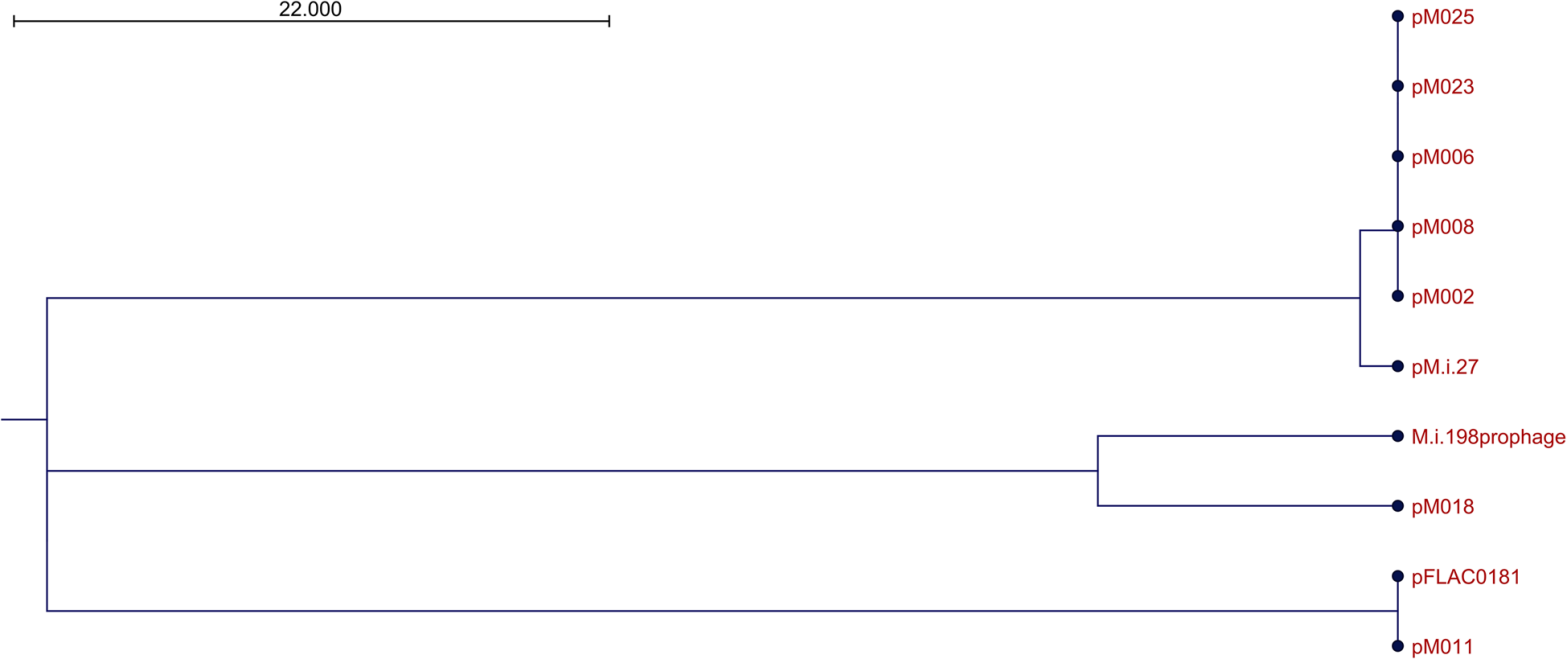
Phylogenetic tree of the plasmids found in this study. The tree was generated based on alignment percentages (AP) using the Neighbor-Joining method

Some *M. intracellulare* strains are known to possess plasmids such as *M. intracellulare* FLAC0181 (plasmid pFLAC0181: NZ_CP023150.1) and *M. intracellulare* subsp. *yongonense* 05–1390 (pMyong1: NC_020275.1, pMyong2: NC_020276.1). In this study, we found a 24 kb plasmid in five strains (M002, M006, M008, M023, M025) in the M.i.27 subgroup and a 26 kb plasmid in the strain M.i.27 (Fig. 2, Fig. S5). These two plasmids were the same, except that the plasmid from M.i.27 (pMi27) possessed a 1.45 kb insertion sequence in the 24 kb plasmid possessed by the other five strains. Some of the genes in these plasmids were annotated and included a MerR family transcriptional regulator, an FAD-dependent pyridine nucleotide-disulfide oxidoreductase, a putative resolvase/invertase/recombinase, and a peptide transporter, but most of the annotated genes were hypothetical. Furthermore, we found that the other 24 kb plasmid in M011 (MP-MIP group) showed high similarity to pFLAC0181 in FLAC0181 (M.i.198 subgroup of TMI group), and a 51 kb plasmid in M018 (TMI group not belonging to the M.i.198, M.i.27, or ATCC13950 subgroups) showed similarity to the M.i.198 prophage sequence that we reported previously [22]. These data suggested that the plasmids in *M. intracellulare* could be regarded as characterizing subspecies, as well as a tool to analyze the evolution of *M. intracellulare* strains to produce new subspecies by the exchange of plasmids between groups of strains.

### Alignment of the genomic regions

**Fig. 3:**
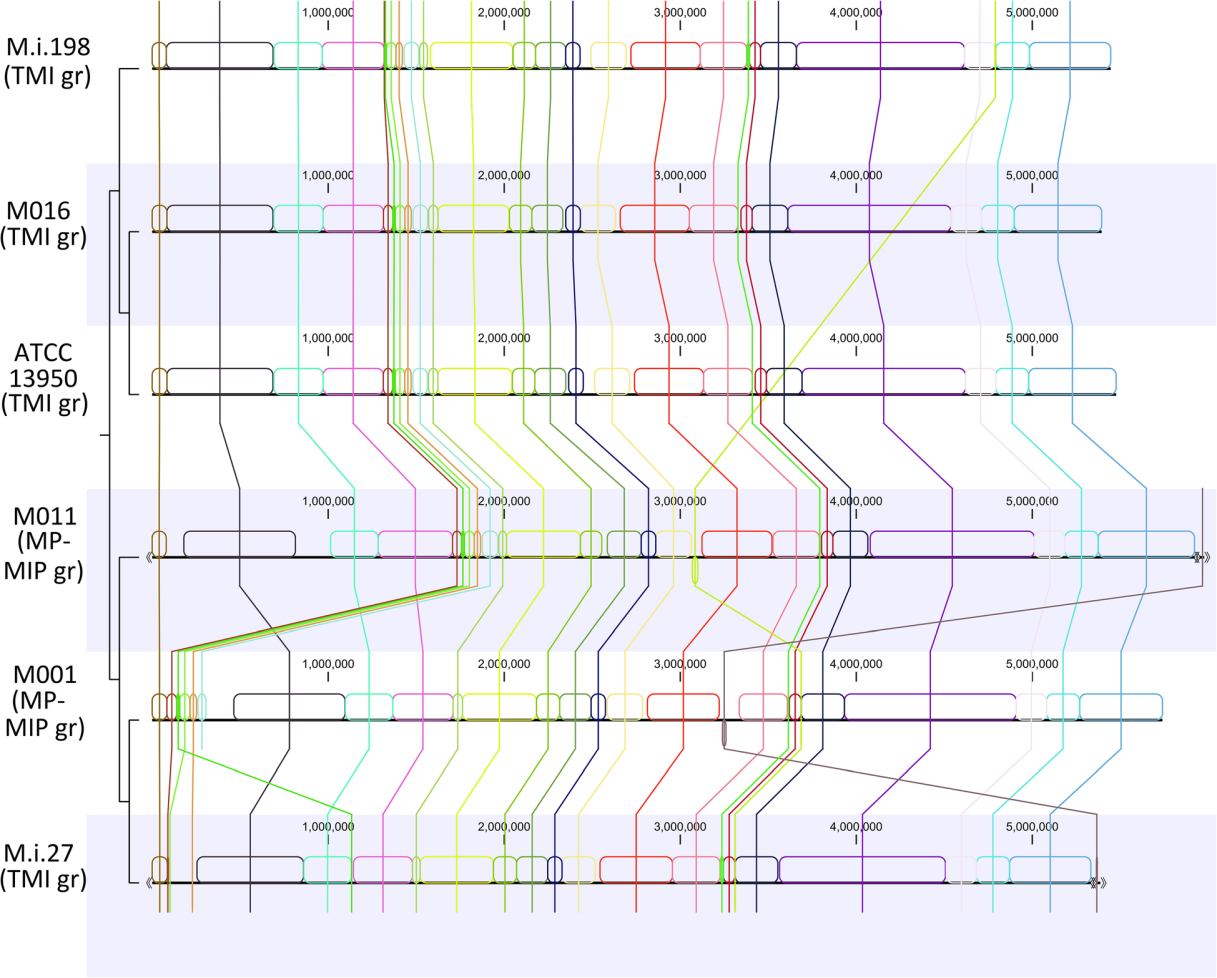
Alignment of genomes of *M. intracellulare* including clinical strains. Data show 40% of the blocks. Several mobile genetic regions were found such as the region from 1330 kb to 1471 kb in ATCC13950, that from 4790 kb to 4791 kb in M.i.198, and that from 15 kb to 16 kb in M011 plasmid

Changes in genomic alignment, such as inversions, are commonly detected among bacteria. Several recent reports revealed genomic inversions in mycobacteria, including *M. avium* [23, 24]. By contrast, genome alignment was generally preserved among *M. intracellulare* species (Fig. 3, Fig. S6). Inversions were detected in mobile regions such as insertion sequences. The inversion of large sequences was found in MIP MTCC9506 (2709 kb – 2968 kb, 3025 kb – 3059 kb, 3114 kb – 3153 kb regions on MTCC9506 genome) as shown previously [16].

### Hypermutated regions

**Fig. 4:**
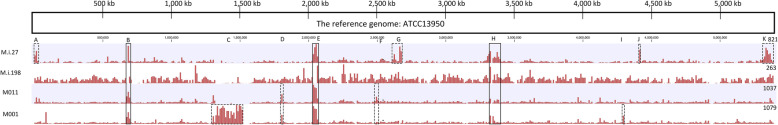
Distribution of single nucleotide variants (SNV) throughout the genome. The detection of SNV was performed using the genome of ATCC13950 as the reference genome. Numbers on the upper right-hand side for each strain indicate the number of SNV as a scale. Regions surrounded by the bold and dashed lines indicate the common regions (**b, e, h**) and group- or subgroup-specific hypermutated regions (**a, c, d, f, g, i, j**), respectively

By setting ATCC13950 as the reference strain, several genomic regions were identified as hot-spots of asynonymous hypermutations (Fig. 4, Table 2, Fig. S7, Table S1). Hypermutated regions (300 mutated sites per 10 kb sequence region) were frequently found, especially among strains in the M.i.27 and MP-MIP groups, some within the same regions and others within different regions. By contrast, hypermutated regions were not found among M.i.198 subgroup strains, with the exception of two consecutive regions in M004 (670 kb – 690 kb regions corresponding to ATCC13950 genome).

**Table 2:** List of the representative genes located in the hypermutated regions

M.i.27 subgroup		
Gene	Locus tag^*^	Function
*gyrA*	OCU_RS25025	DNA gyrase subunit A
*cyp144*	OCU_RS25050	cytochrome P450 Cyp144
*cwsA*	OCU_RS25055	cell wall synthesis protein CwsA
*citE*	OCU_RS37365	citrate lyase subunit beta
*zwf*	OCU_RS37375	IMP dehydrogenase
*hrpA*	OCU_RS37390	ATP-dependent RNA helicase
*otsB*	OCU_RS45365	trehalose-phosphate phosphatase
*htdY*	OCU_RS45370	3-hydroxyacyl-thioester dehydratase HtdY
*lpqD*	OCU_RS45375	histidine phosphatase family protein
*eccC2*	OCU_RS49810	type VII secretion protein EccC
*mviN*	OCU_RS49890	putative peptidoglycan biosynthesis protein MviN
*rsmA*	OCU_RS49900	anti-sigma-M factor RsmA
*trxB_2*	OCU_RS49905	thioredoxin reductase
**MP-MIP group**		
**Gene**	Locus tag^*^	**Function**
*paaG*	OCU_RS31460	enoyl-CoA hydratase
*mce3A*	OCU_RS31625	MCE-family protein Mce3A
*mce3B*	OCU_RS31630	Mce family protein Mce3B
*mce3C*	OCU_RS31635	Mce family protein Mce3C
*mce3D*	OCU_RS31640	Mce family protein Mce3D
*lprM*	OCU_RS31645	MCE-family protein Mce3E
*hmd*	OCU_RS31690	5,10-methylene tetrahydromethanopterin reductase
*cyp143*	OCU_RS31715	putative cytochrome P450 Cyp143
*fadE3*	OCU_RS31935	acyl-CoA dehydrogenase
*acd*	OCU_RS32010	acyl-CoA dehydrogenase
*fadE33*	OCU_RS32080	acyl-CoA dehydrogenase
*cyp124*	OCU_RS32180	methyl-branched lipid omega-hydroxylase
*mhpB*	OCU_RS32235	2,3-dihydroxyphenylpropionate/2,3-dihydroxicinnamic acid 1,2-dioxygenase
*cyp143*	OCU_RS32255	putative cytochrome P450 Cyp143
*fadE22*	OCU_RS32275	acyl-CoA dehydrogenase FadE22
*mmpL13*	OCU_RS33545	Transport protein MmpL13
**Both M.i.27 subgroup and MP-MIP group**		
**Gene**	Locus tag^*^	**Function**
*mmpL12*	OCU_RS28435	putative transport protein MmpL12
*cyp123*	OCU_RS28470	putative cytochrome P450 Cyp123
*acg*	OCU_RS34620	putative NADPH nitroreductase Acg

^*^Locus tag assigned on ATCC13950

The genes of a mammalian cell entry (*mce*) operon (*mce3ABCD-lprM*) [25, 26] and those involved in fatty acid metabolism (*acd2, fadE3, fadE22, fadE33*), a folate synthase (*hmd*) and those encoding cytochrome P450 (*cyp124, cyp143*) were included in the hypermutation regions identified in the clinical strains. The nitroreductase gene (*acg*) was commonly found in the hypermutated regions in the strains belonging to the groups M.i.27 and MP-MIP. Genes encoding a transporter (*mmpL12*) and cytochrome P450 (*cyp123*) were found in the hypermutated regions of some strains belonging to the M.i.27 subgroup and the MP-MIP group. Genes involved in DNA and RNA metabolism (*gyrA*, *hrpA*), the phosphatase of trehalose and tyrosine (*otsB, lpqD*), an immune-regulating protein (*htdY*) [27], a dehydrogenase (*zwf*), cell wall metabolism (*cwsA, mviN*), and a type VII secretion system (*eccC2*) were included in the hypermutated regions in the strains of the M.i.27 subgroup. A trehalose glycolipid transporter gene (*mmpL13*) was found in the hypermutated region in most strains of the MP-MIP group. However, the location of the other hypermutated regions varied from strain to strain. The genes located in hypermutated regions were functionally consistent with the evolution of mycobacteria-specific metabolism, such as the synthesis of mycolic acids and trehalose glycolipids, as well as long-term persistence in the host.

### Pan-genomic analysis

**Fig. 5:**
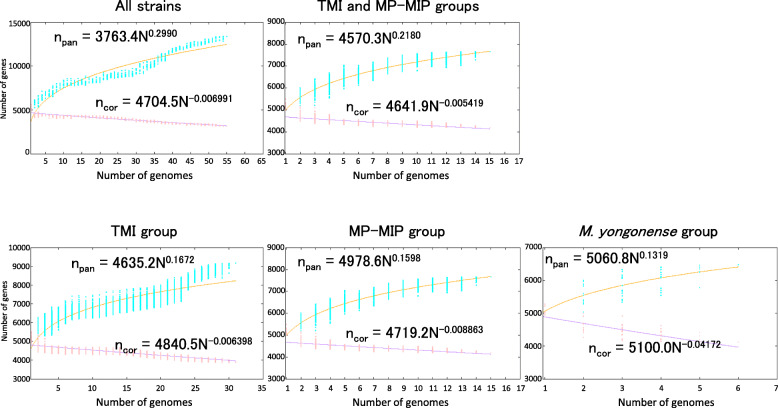
Pan and core genome analysis. Power law regression curves were generated based on the numbers of pan and core genes calculated for 200 iterations

Pan-genomic analysis was performed to calculate the genomic diversity of *M. intracellulare* strains including strains belonging to the TMI, MP-MIP, and *M. yongonense* groups. A prominent feature was that the proportion of accessory genes to core genes (approximately 1900 accessory genes and 3153 core genes) was comparatively higher in the strains enrolled in this study than in *M. tuberculosis* clinical strains (approximately 550 accessory genes and 3679 core genes) (Table S2) [7]. Furthermore, some strains possessed hundreds of unique genes, while others possessed only a few unique genes. Strains possessing a large number of unique genes were dispersed among all subspecies and were not limited to a particular subspecies. When considering the accumulation of new genes in these strains, the exponent of the number of pan-genes expressed by the *γ* parameter from Heaps’ law was greater than zero (0.2990) and *M. intracellulare* had 11,513 pan-genes (*n* = 55) indicating an open pan-genome [28] (Fig. 5). At the level of groups of strains, the exponent was decreased but equivalent to that reported for *M. avium* (γ = 0.1935) indicating an open pan-genome among the groups of strains.

To determine whether the absence of particular genes amongst groups of strains is of value in determining strain-specific virulence factors. In this study, we identified 151, 421, and 766 clusters of exclusively-absent genes in the groups of TMI, MP-MIP, and *M. yongonense*, respectively, from the total of 290,267 input genes generated by pan-genomic analysis using Bacterial Pan-Genome Analysis tool (BPGA) software [29]. Of note, with the exception of hypothetical genes, the gene family most commonly associated with exclusively absent genes was the *mce* gene family (Table 3). Mce functions as a possible ATP-binding cassette transporter in mycobacteria and the number of operons and the levels of sequence similarity among *mce* genes are diverse among mycobacteria [25, 26]. In *M. intracellulare* ATCC13950, the *mce1* and *mce4* operons showed high similarity with those of *mce1*_*Mtb*_ and *mce4*_*Mtb*_ (average 80% identity) (Table S3). Two *mce3* operons existed that showed lower sequence similarity with those of *mce3*_*Mtb*_ (average 65% identity) and additional three operons existed that also showed low sequence similarity with those of various types of *mce*_*Mtb*_ genes (20–40% identity). These three operons were phylogenetically grouped into *mce7*, *mce7bis* and *mce5* of *M. smegmatis* (Fig. S8). By comparing the sequences of the identified, exclusively-absent, *mce* genes with those of *mce* sequences in *M. tuberculosis*, *M. smegmatis*, and *M. intracellulare* ATCC13950, the composition of *mce* operons was suggested to be group-specific as follows: i) There was an additional *mce5* operon in the strains of the MP-MIP group and *M. yongonense* not found in those of the TMI group. ii) There was an additional *mce1* operon in the strains of the TMI group and *M. yongonense* not found in those of the MP-MIP group. iii) The *mce3* operons were present only in the strains of the groups of TMI and MP-MIP not found in those of *M. yongonense* (Table 3, Fig. S9).

**Table 3:** Group specificity of the *mce* genes in terms of the detection of the exclusively absent clusters of genes by pan-genomic analysis

Absent from TMI group		
**Derived strain (strain group) of hit cluster**	**Possible function**	**Homologous genes in ATCC13950**
MOTT64(MP-MIP)	virulence factor Mce	OCU_RS29085
MOTT64(MP-MIP)	mammalian cell entry protein	OCU_RS29065
KCTC29084(MP-MIP)	mammalian cell entry protein	OCU_RS29070
JCM30622(MP-MIP)	mammalian cell entry protein	OCU_RS29075
JCM30622(MP-MIP)	virulence factor Mce family protein	OCU_RS29060
1099801.4(*M. yongonense*)	virulence factor Mce	OCU_RS29080
**Absent from MP-MIP group**		
**Derived strain (strain group) of hit cluster**	**Possible function**	**Homologous genes in ATCC13950**
M023(TMI)	mammalian cell entry protein	OCU_RS48855
FLAC0162(TMI)	Mce family protein Mce1B	OCU_RS48875
1099801.4(*M. yonogonense*)	Mce family protein Mce1C	OCU_RS48870
05–1390(*M. yonogonens*e)	Mce family protein Mce2A	OCU_RS28225
05–1390(*M. yonogonense*)	Mce family protein Mce1D	OCU_RS48865
**Absent from** ***M. yongonense*** **group**		
**Derived strain (strain group) of hit cluster**	**Possible function**	**Homologous genes in ATCC13950**
M021(MP-MIP)	mammalian cell entry protein	OCU_RS31630
M.i.27(TMI)	Mce family protein Mce3B	OCU_RS32530
M023(TMI)	mammalian cell entry protein	OCU_RS32545
M025(TMI)	Mce family protein Mce3D	OCU_RS31640
M025(TMI)	Mce associated membrane protein	OCU_RS32555
M026(TMI)	mammalian cell entry protein	OCU_RS31650
M028(TMI)	Mce family protein Mce3D	OCU_RS32540
M010(TMI)	Mce family protein Mce3C	OCU_RS32535
M017(MP-MIP)	Mce family protein Mce1D	OCU_RS48865
M017(MP-MIP)	mammalian cell entry protein	OCU_RS48860
M017(MP-MIP)	Mce associated protein	OCU_RS48840
M012(MP-MIP)	mammalian cell entry protein	OCU_RS32550
CSURP8077(TMI)	Mce family protein Mce1B	OCU_RS48875
CSURP8077(TMI)	Mce family protein Mce1C	OCU_RS48870
CSURP8077(TMI)	mammalian cell entry protein	OCU_RS48855
2285(TMI)	Mce family protein Mce3C	OCU_RS31635
1956(TMI)	MCE-family protein MCE3A	OCU_RS31625
FLAC0181(TMI)	Mce associated membrane protein	OCU_RS31655

## Discussion

In this study, we revealed that clinical *M. intracellulare* strains could be grouped into two distinct groups, namely typical *M. intracellulare* (TMI) and MP-MIP groups, by comparative genomic analysis. Our data were consistent with the previous whole genome sequencing data of a representative strain from each nontuberculous mycobacterial species claiming that *M. paraintracellulare* should be reclassified into *M. intracellulare* at the subspecies level (average nucleotide identity ≥98%, alignment percentage ≥ 80%) [30–32]. Furthermore, we demonstrated that *M. indicus pranii* MTCC9506 was not classified into the TMI group but into the MP-MIP group. Thus, we propose that the groups of TMI and MP-MIP are reclassified as *M. intracellulare* subsp. *intracellulare* genovar *intracellulare* (including the type strain ATCC13950) and genovar *paraintracellulare* (including the type strain MOTT64), respectively. In this context, we also propose that *M. indicus pranii* MTCC9506 is in fact a strain of *M. intracellulare* subsp. *intracellulare* genovar *paraintracellulare*.

Comparative genomic approaches by whole genome sequencing have arisen the reconsideration of the classification of some *M. intracellulare*-related strains. The *M. yongonense* strains and *M. intracellulare* strains enrolled in this study showed synonymous similarity level in the species rank (average nucleotide identity ≥96%, alignment percentage ≥ 70%), which supports the claim that *M. yongonense* should be reclassified into *M. intracellulare* subsp. *yongonense* [30–33]. *M. chimaera* was proposed as a new species of MAC in 2004 from the characteristics of the sequences of 16Sr RNA and 16S–23S internal transcribed spacer as well as the unique mycolic acid pattern [34]. *M. chimaera* seems to have some difference in etiological background from other species causing pulmonary MAC disease because of the reported outbreak in the open-heart surgery patients from the contaminated heater-cooler units [35]. Despite such distinct clinical features, *M. chimaera* has been claimed to be taxonomically synonymous with *M. intracellulare* at the species or subspecies levels (average nucleotide identity ≥96%, alignment percentage 79–82%) [19, 30–32]. Similar to our study, comparative genomic analyses of a sufficient number of clinical strains may be able to verify the claim.

There are several factors causing taxonomic controversies in *M. intracaellulare* and related strains. The first is the method of defining the bacterial species. Compared to the DNA-DNA hybridization techniques which is still regarded as the gold standard method for analyzing genomic similarities, computational calculation of similarity indices such as average nucleotide identity and alignment percentage provides robust and reproducible data for taxonomic classification. For example, DNA-DNA hybridization value was reported to be 53% between *M. paraintracellulare* MOTT64 and *M. intracellulare* ATCC13950 in contrast to the computational indices suggesting synonymous subspecies between the two (average nucleotide identity > 98%, predicted DNA-DNA hybridization value calculated from genome blast distance phylogeny > 80%) [13]. And the genomic similarity has been verified by the following whole genome sequencing data including ours [19, 31, 32]. The second is which strain is chosen as a representative strain from each species for comparative genomic analyses. Our data were consistent with the study by Tortoli showing that *M. intracellulare* and *M. paraintracellulare* are monophyletic and *M. yongonense* is paraphyletic to *M. intracellulare* and *M. paraintracellulare* [19]. On the other hand, our data were different from the study by Matsumoto showing that *M. indicus pranii* and *M. intracellulare* are monophyletic but *M. intracellulare* and *M. paraintracellulare* are paraphyletic [20]. The former study chose *M. intracellulare* ATCC13950, a type strain of TMI, but the latter study chose *M. intracellulare* MIN_052511_1280, a miscellaneous strain neither belonging to the TMI group nor the MP-MIP group. The different choice of the strains enrolled for analyses causes taxonomic discrepancies. Comparative genomic analysis enrolling a sufficient number of clinical strains is considered to be an important step in taxonomic studies. The third is the relationship between genomic similarities and other clinical features including phenotypes and epidemiology. Based on the genomic similarity data, some species within the *M. tuberculosis* complex (i.e. *M. africanum*, *M. bovis*, *M. caprae*, *M. microti* and *M. pinnipedii*) have been reclassified as *M. tuberculosis* [36]. Similar to the case of *M. tuberculosis*, *M. yonogonense* and *M. chimaera* have been proposed to be reclassified as synonymous with each other at the species or subspecies levels [19, 30–32]. Such reclassification may increase the species and subspecies categories that include mycobacteria with distinct clinical features. How to integrate the information of clinical features into the phylogenetic data based on genomic similarities should be further investigated for consistent taxonomic classification and nomenclature between the fields of bacteriology and clinical infectious disease.

Previous studies using multilocus sequence typing of housekeeping genes demonstrated the obscure classification of TMI strains with distinct sub-grouping between MP and MIP [18, 37]. We found that the subgroups of M.i.198 (including MOTT-02) and ATCC13950 were barely classified by multilocus sequence typing but these were phylogenetically very close (Fig. S10). The strains of the MP-MIP subgroup were distinctly classified into the two groups (one included *M. paraintracellulare* MOTT64 and the other included *M. indicus pranii* MTCC9506). However, the branching pattern of each strain was different from the genomic phylogeny. The strains of the M.i.27 subgroup were not able to be classified by the sequence-based genotyping because they were placed on the root of the phylogenic trees. Taken together, whole genome sequencing method should be recommended for modernizing clinical taxonomy.

Several types of plasmids were detected among clinical *M. intracellulare* strains. Plasmid pMI27 and its related plasmids found in the M.i.27 subgroup were novel. However, pMI27 was not detected in every strain belonging to the M.i.27 subgroup, suggesting heterogeneity in their plasmid profiles. The identification of similar plasmids among the different groups and subgroups of strains, such as pFLAC0181 in M011 and M.i.198 prophage in M018, seems to infer the possible transmission of the sequence during strain evolution.

Mycobacteria are characterized by their unique lipid metabolism involving the synthesis of long-chain fatty acids such as mycolic acids. The inclusion of several genes of fatty acid metabolism (*fadE3, fadE33*) in the hypermutation hot-spot regions suggested the adaptation to survive in the host. Similarly, the inclusion of genes encoding lipid transporters (*eccB2, eccC2, mce3* operon) in the hypermutation hot-spot regions in the strains of the M.i.27 subgroup and the strains of the MP-MIP group suggested adaptation in the uptake of lipids to ensure survival in the host [38].

By including the data for clinical strains, *M. intracellulare* was proven to be pan-genomic, similar to other mycobacteria, such as *M. avium* and *M. tuberculosis* [6, 7, 9]. The comparatively higher proportion of non-core genes to core genes in *M. intracellulare* suggested higher genomic evolutionary activity than in *M. tuberculosis*, which may be associated with the diversity of bacteriological characteristics (colony appearance, growth rate in medium, and experimental virulence) and clinical manifestations [21, 39–41]. In this study, the *mce* operons were found to be a group-specific gene family. Mce proteins constitute an ABC transporter in mycobacteria thought to transport phospholipids, based on homology to the Mce4 system in *M. tuberculosis* that transports cholesterol and is required for persistent in vivo infection [42]. The *mce1* operon has been suggested to be a mycolic acid re-importer [43] but the function of the other *mce* operons has not been elucidated. Identifying the *mce* operons as group-specific genes is an important step in elucidating the virulence factors in *M. intracellulare* and its related strains. In particular, the *mce1* operon seems to be a promising candidate of virulence factors with evidence of its role in hypoxic biofilm formation, as revealed by the genome-wide identification of essential genes by transposon sequencing [44].

In *M. yongonense*, the lateral gene transfer event has been suggested in *rpoB* gene from a distantly related scotochromogen *M. parascrofulaceum* by multi-locus sequence typing [45]. Taking this notion into consideration, the accumulation of lateral gene transfer events may explain the incongruence of the phylogenetic trees based on limited housekeeping genes and those based on genomic sequences. On the other hand, in genomic level, the impact of lateral gene transfer on genomic evolution has been estimated to be low in mycobacteria because the percentage of genomes resulting from lateral gene transfer is low (ranging from 0.04 to 1.9%) without large recombination (> 1 kb) [46]. To overcome the complexity of the taxonomic classification by the lateral gene transfer, the prior use of whole genomic sequencing data should be recommended for discussing genomic features of clinical strains, rather than the simple use of genotyping data of housekeeping genes.

Our data provide basic information on the genetic similarity and diversity of clinical *M. intracellulare* strains that enables a better understanding of the evolution of strains causing pulmonary MAC disease. However, this study has some limitations. The population size of this study was small and there were some strains that belonged outside of the TMI or MP-MIP groups (E2190, E3191) and did not belong to the major subgroups of M.i.27, ATCC13950, and M.i.198. Characterization of these orphan strains may be of value to fully elucidate the relationship between genomic differences and pathogenic phenotypes in pulmonary MAC disease. The typing of genome sequences of a collection of Japanese clinical isolates is ongoing. The addition of newly isolated strains from *M. intracellulare* endemic areas, such as the US and India, may also give some insight into the genomic characteristics of this pathogen globally [2, 4]. The biological significance of the group-specific genes identified by comparative genomics remains to be determined. In contrast to *M. tuberculosis*, biological experiments using deletion mutants have not been reported for pathogenic mycobacteria, with the exception of *M. marinum* that mimics *M. tuberculosis* as it possesses the ESX-1 secretion system [47]. We have recently modified a gene manipulation method for *M. intracellulare* using mycobacterial plasmids and phages developed for *M. tuberculosis* [44]. The next step will be to analyze the biological effects in these deletion mutants to fully elucidate the molecular mechanisms of pulmonary MAC disease.

## Conclusions

We have clarified the major groups of *M. intracellulare* by comparative genomic analysis of the clinical strains derived from pulmonary MAC disease patients and previously-reported genome sequences. Furthermore, we have clarified the diversity of *M. intracellulare* by pan-genomic analysis and the *mce* operons were identified as contributing to the genomic diversity. These data provide vital genetic information that can be used to elucidate the virulence factors and mechanisms of pathogenesis in recently-emerged non-tuberculous mycobacterial disease.

## Methods

### Study subjects

A total of 29 non-HIV patients with pulmonary MAC disease were enrolled in this study between January 2015 and March 2019. All patients were either treated as outpatients or were hospitalized in the National Hospital Organization Osaka Toneyama Medical Center. This study was approved by the Institutional Review Board of the National Hospital Organization Osaka Toneyama Medical Center and Niigata University Hospital. The opportunity to opt-out of consent was provided for all participants. The diagnosis of MAC disease was conducted based on the diagnostic guidelines proposed by the American Thoracic Society [39, 40]. The diagnosis of clinical specimens was performed by a DNA-DNA hybridization assay using AccuProbe (Gen-Probe Inc., San Diego, CA) or COBAS AMPLICOR (Roche Diagnostic, Tokyo, Japan) systems or by DNA-DNA hybridization assay (Kyokuto Pharmaceutical Industrial, Tokyo, Japan). After treatment with NaOH and N-acetyl-L-cysteine, the sputum samples were streaked onto Lowenstein–Jensen medium. Single colonies were isolated on 7H10/OADC agar plates. One isolated strain per patient was analyzed in this study. Two additional clinical *M. intracellulare* strains M.i.198 and M.i.27, which had previously been reported to be virulent in mice, were included in this study [21]. Genome sequence data for strains registered by other researchers in the NCBI database were obtained and used in this study.

### Genome sequencing

Genomic DNA from the clinical strains was isolated by the phenol-chloroform method as previously described [48]. Sequencing was performed using the MiSeq system and PacBio RS-II. Sequencing libraries for MiSeq were prepared using the Nextera XT DNA Library Preparation Kit according to the manufacturer’s protocol (Illumina, CA). Each DNA library with adapters was normalized to 4 nM, pooled, and sequenced by the MiSeq system with MiSeq Reagent Kit v3 (Illumina, CA). Sequencing libraries for PacBio were prepared using SMRTbell Template Prep Kit 1.0 according to the manufacturer’s protocol (Pacific Biosciences, CA). For each SMRTbell DNA library, size selection was performed using BluePippin. Each size selected SMRTbell DNA library was sequenced by the PacBio RS-II system with DNA Sequencing Reagent Kit 4.0 v 2 (Pacific Biosciences, CA). All genome sequences, including the strains registered by other researchers in NCBI, were annotated using dFAST to standardize the data prior to pan-genomic analysis [49].

### Analysis of genome sequence data

Genome sequence data were handled by the CLC Genomics Workbench system (Qiagen Inc., Valencia, CA). Species identification was performed based on the genome data by finding the best match species from the NCBI bacteria database (downloaded on June 25, 2020). A complete genome sequence was obtained by mapping the reads obtained by MiSeq to those obtained by PacBio and the consensus sequence was adopted as the complete genome sequence if the sequence could be finished as a circular chromosome with/without a circular plasmid. *M. intracellulare* ATCC13950 was set as the reference strain. The whole genome sequences were aligned using the analytic tool provided by the CLC Genomics Workbench system, with the following default settings: minimum initial seed length 15, allow mismatches in seeds, minimum alignment block length 100. The identification of asynonymous single nucleotide polymorphisms was performed using the analytic tools provided by the same Workbench system.

Pan-genomic analysis, including the identification of exclusively-absent genes in the designated groups of strains, was performed using Bacterial Pan-Genome Analysis tool (BPGA) software [29]. Clustering of the genes was performed using USEARCH on BPGA with cut-off sequence identity set at 80%. Pan-genome profile calculations were performed with 200 iterations of combinations.

## Supplementary Information

**Additional file 1.**

**Additional file 2.**

**Additional file 3.**

**Additional file 4.**

## Data Availability

The datasets generated in this study are deposited in the DNA Data Bank of Japan (DDBJ) (https://ddbj.nig.ac.jp/), Accession Number: DRA011116. The data deposited is publicly available.
