# Supplementary material for: Comparative genomic analysis of Mycobacterium intracellulare: implications for clinical taxonomic classification in pulmonary Mycobacterium avium-intracellulare complex disease
Source: BMC Microbiol. 2021 Apr 6;21:103. doi: 10.1186/s12866-021-02163-9 (PMC8025370; doi:10.1186/s12866-021-02163-9)
Supplement: Supplementary file 1 — Additional file 1. [file 12866_2021_2163_MOESM1_ESM.zip › 20201001SupplFigS3.pptx]

## Slide 1
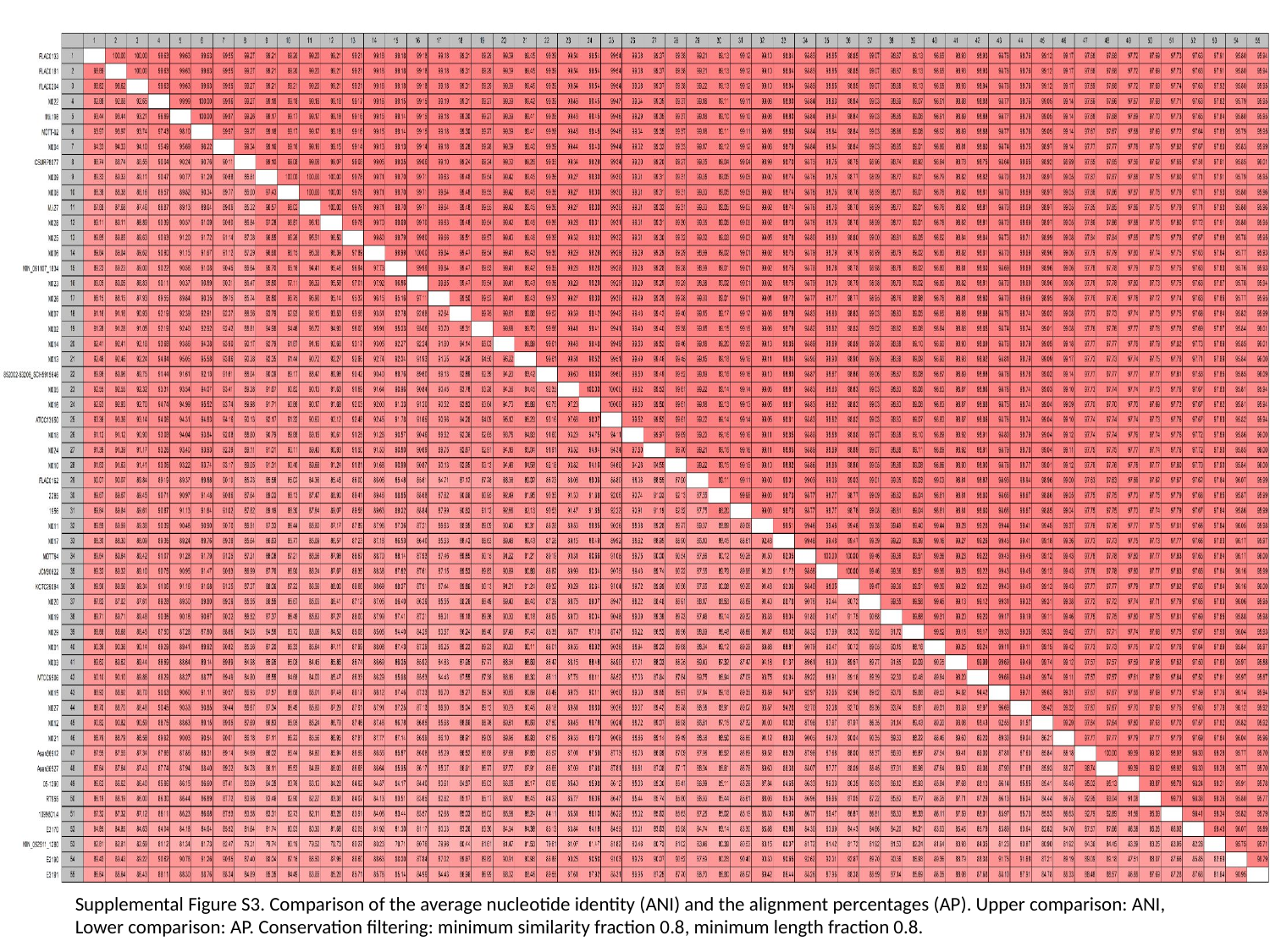

Supplemental Figure S3. Comparison of the average nucleotide identity (ANI) and the alignment percentages (AP). Upper comparison: ANI, Lower comparison: AP. Conservation filtering: minimum similarity fraction 0.8, minimum length fraction 0.8.
