# Supplementary material for: Comparative genomic analysis of Mycobacterium intracellulare: implications for clinical taxonomic classification in pulmonary Mycobacterium avium-intracellulare complex disease
Source: BMC Microbiol. 2021 Apr 6;21:103. doi: 10.1186/s12866-021-02163-9 (PMC8025370; doi:10.1186/s12866-021-02163-9)

Supplementary file section

Comparative genomic analysis of *Mycobacterium intracellulare*: Implications for clinical taxonomic classification in pulmonary *Mycobacterium avium-intracellulare* complex disease

Yoshitaka Tateishi, Yuriko Ozeki, Akihito Nishiyama, Mari Miki, Ryoji Maekura, Yukari Fukushima, Chie Nakajima, Yasuhiko Suzuki, Sohkichi Matsumoto

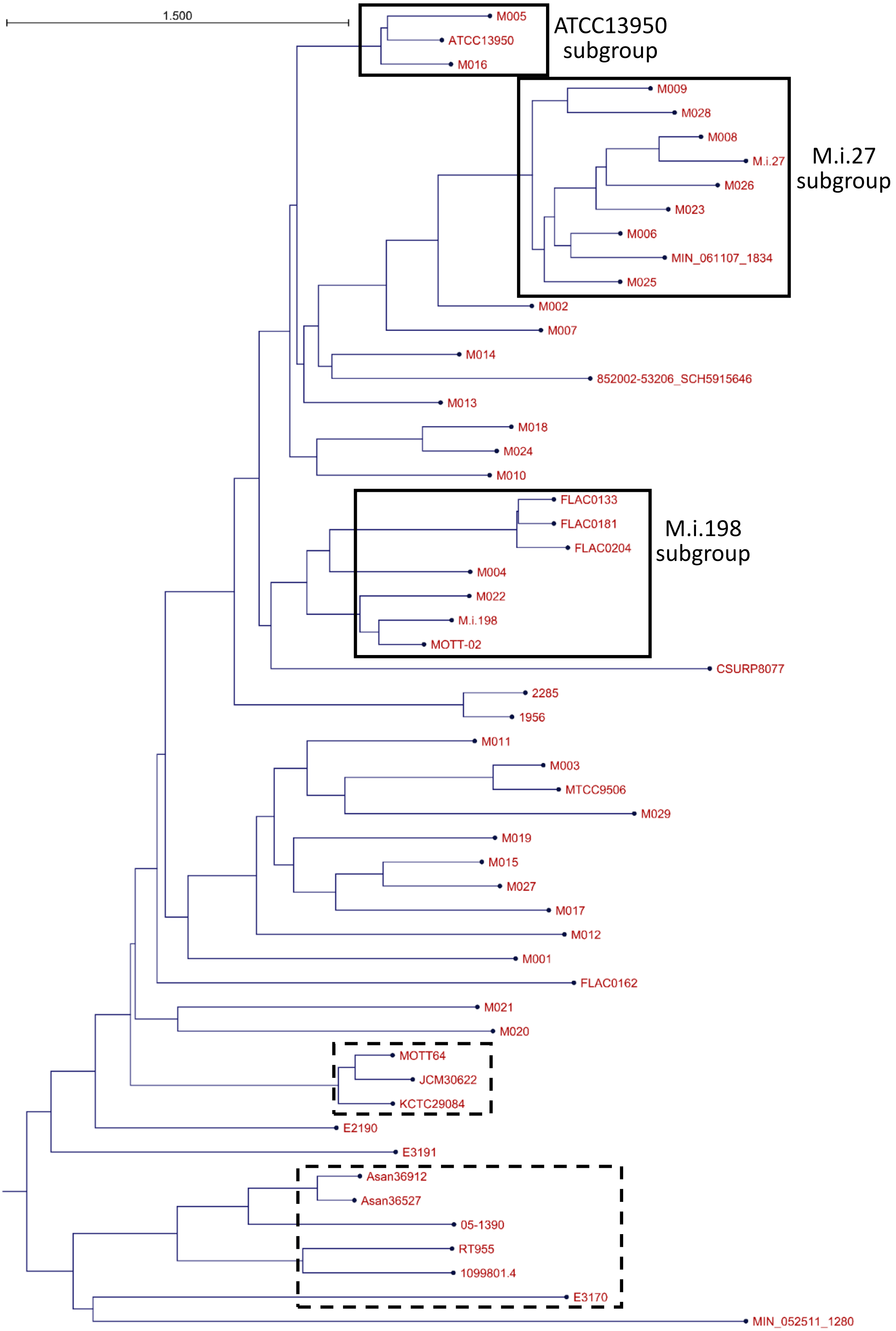

Supplemental Figure S1. Phylogenetic tree of clinical *M. intracellulare* generated based on alignment percentage (AP) using the Neighbor-Joining method. Strains in broken squares indicate those of *M. paraintracellulare* and *M. yongonense* that have been previously registered in the NCBI database.

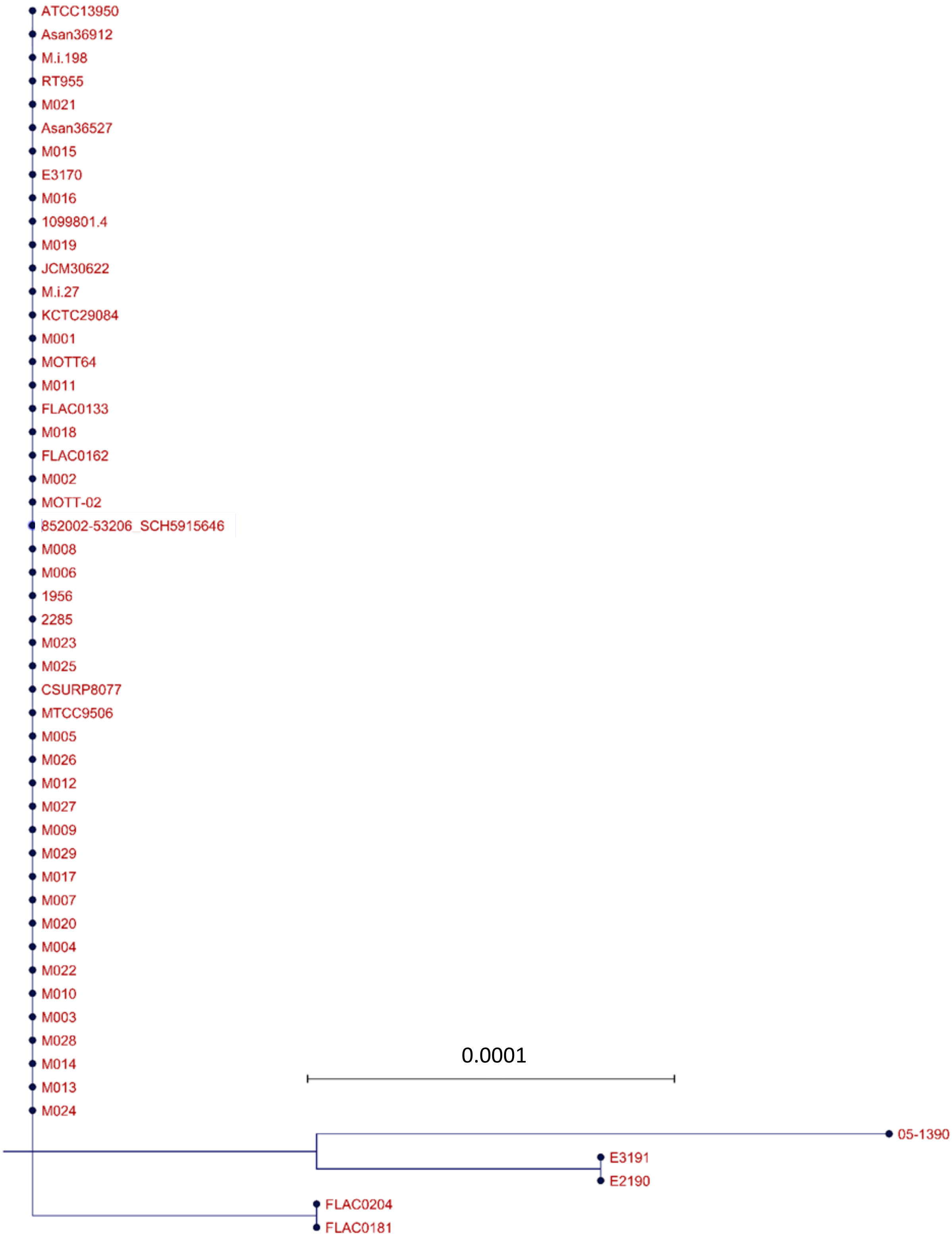

Figure S2. Phylogenetic tree of *M. intracellulare* strains based on the comparison of their 16SrRNA sequences. The tree was generated by using the Neighbor-Joining method.

Figure S4
(A)

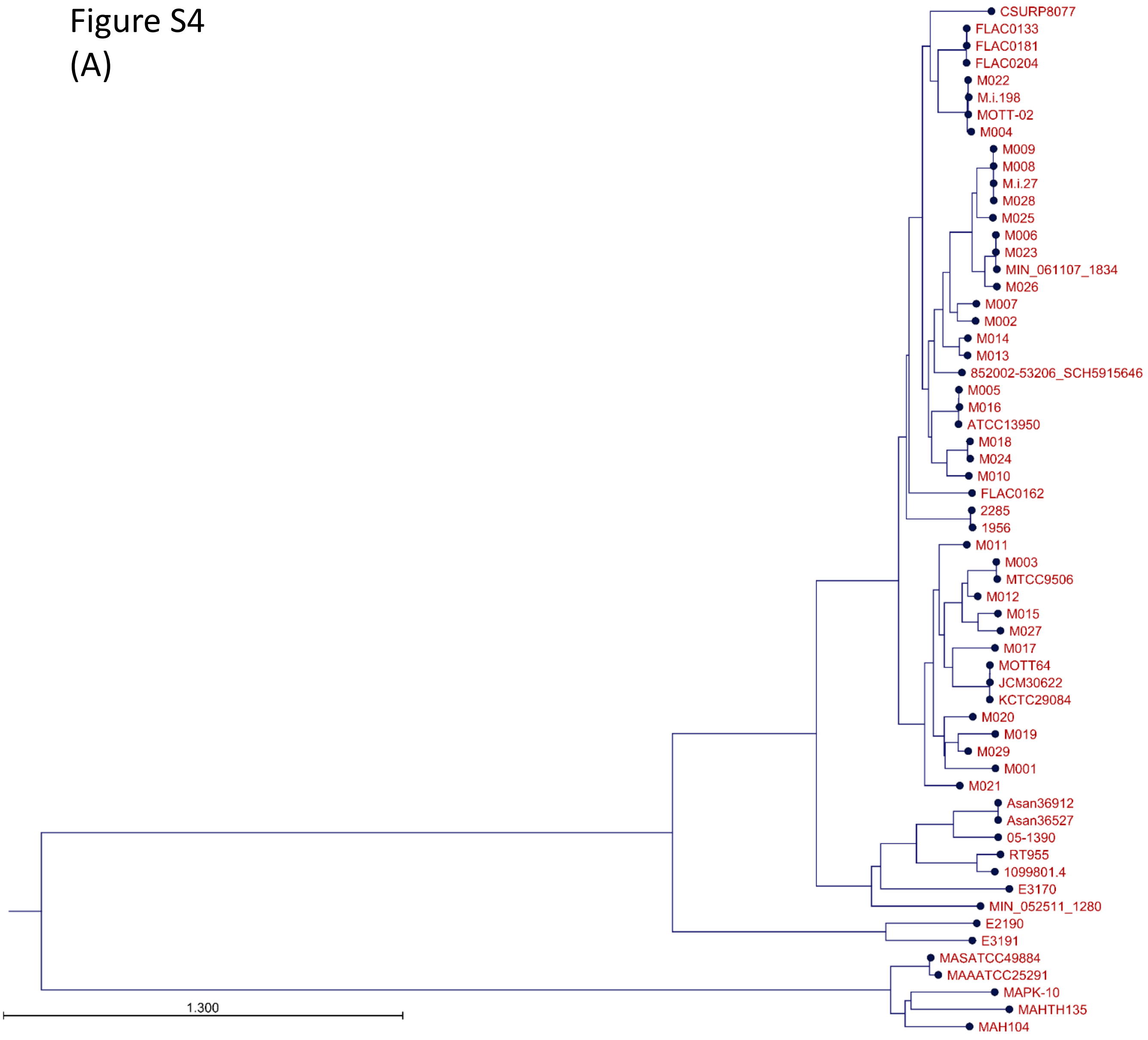

Supplemental Figure S4. Phylogenetic tree of clinical *M. intracellulare* strains and 5 representative *M. avium* strains (*M. avium* subsp. *silvaticum* ATCC49884 [GenBank accession number: AYOC01], *M. avium* subsp. *avium* ATCC25291 [ACFI01], *M. avium* subsp. *paratuberculosis* K-10 [NC_002944.2], *M. avium* subsp. *hominissuis* TH135 [NZ_AP012555.1 and NZ_AP012556.1], *M. avium* subsp. *hominissuis* 104 [NC_008595.1]) . (A) Phylogenetic trees generated based on average nucleotide identity (ANI) using the Neighbor-Joining method. (B) Phylogenetic trees generated based on alignment percentage (AP) using the Neighbor-Joining method.

Figure S4 (Continued)
(B)

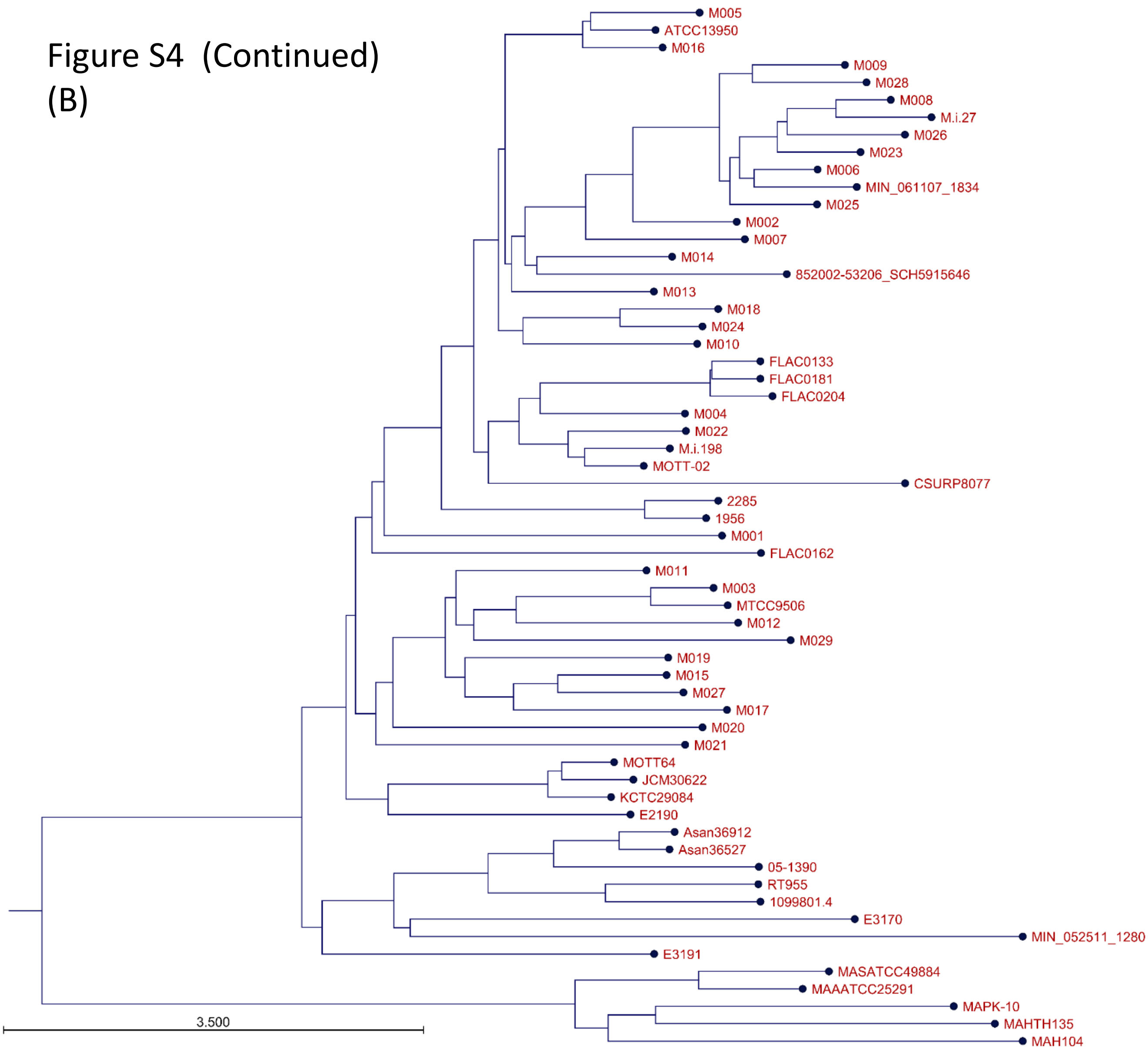

A.

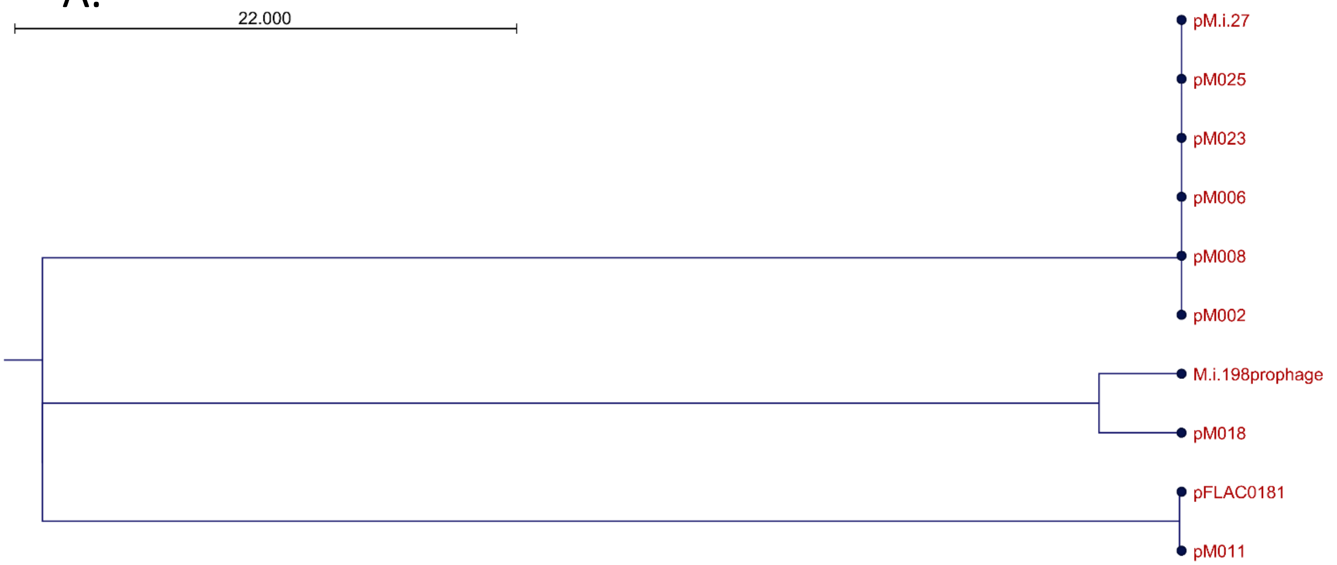

B.

		1	2	3	4	5	6	7	8	9	10
pM.i.27	1		100.00	100.00	100.00	100.00	100.00	0.00	0.00	0.00	0.00
pM025	2	97.21		100.00	100.00	100.00	100.00	0.00	0.00	0.00	0.00
pM023	3	97.21	100.00		100.00	100.00	100.00	0.00	0.00	0.00	0.00
pM006	4	97.21	100.00	100.00		100.00	100.00	0.00	0.00	0.00	0.00
pM008	5	97.21	100.00	100.00	100.00		100.00	0.00	0.00	0.00	0.00
pM002	6	97.21	100.00	100.00	100.00	100.00		0.00	0.00	0.00	0.00
pM011	7	0.00	0.00	0.00	0.00	0.00	0.00		99.83	0.00	0.00
pFLAC0181	8	0.00	0.00	0.00	0.00	0.00	0.00	100.00		0.00	0.00
pM018	9	0.00	0.00	0.00	0.00	0.00	0.00	0.00	0.00		92.75
M.i.198prophage	10	0.00	0.00	0.00	0.00	0.00	0.00	0.00	0.00	77.79	

Supplemental Figure S5. (A) Phylogenetic tree of the plasmids found in this study. The tree was generated based on the average nucleotide identity (ANI) scores using the Neighbor-Joining method. (B) Comparison of the similarity of the plasmids found in this study. Upper comparison: average nucleotide identity (ANI), Lower comparison: alignment percentages (AP). The calculation was performed using the Neighbor-Joining method.

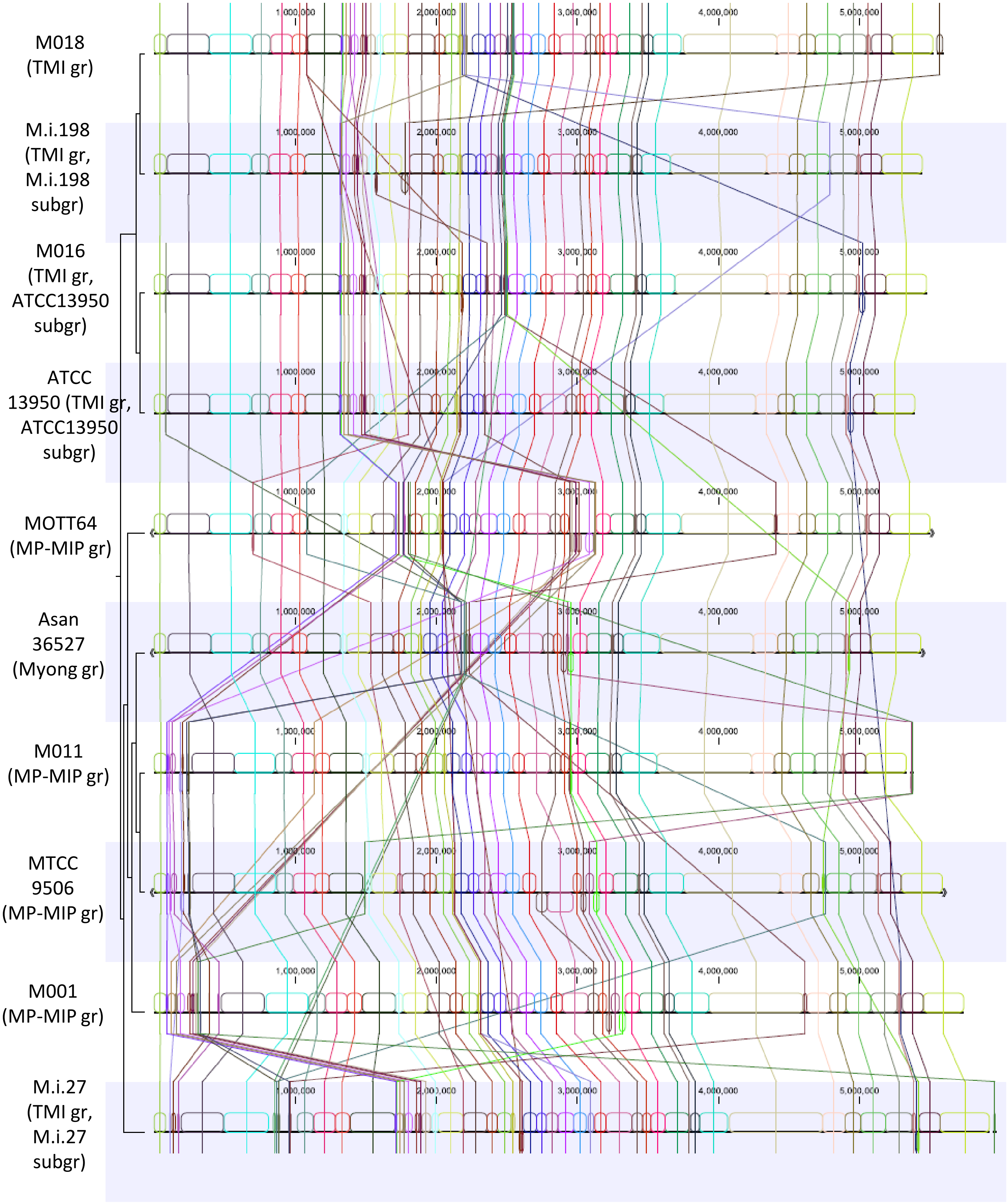

Supplemental Figure S6. Alignment of the genomes of *M. intracellulare* including clinical strains. Data are shown 74% of blocks.

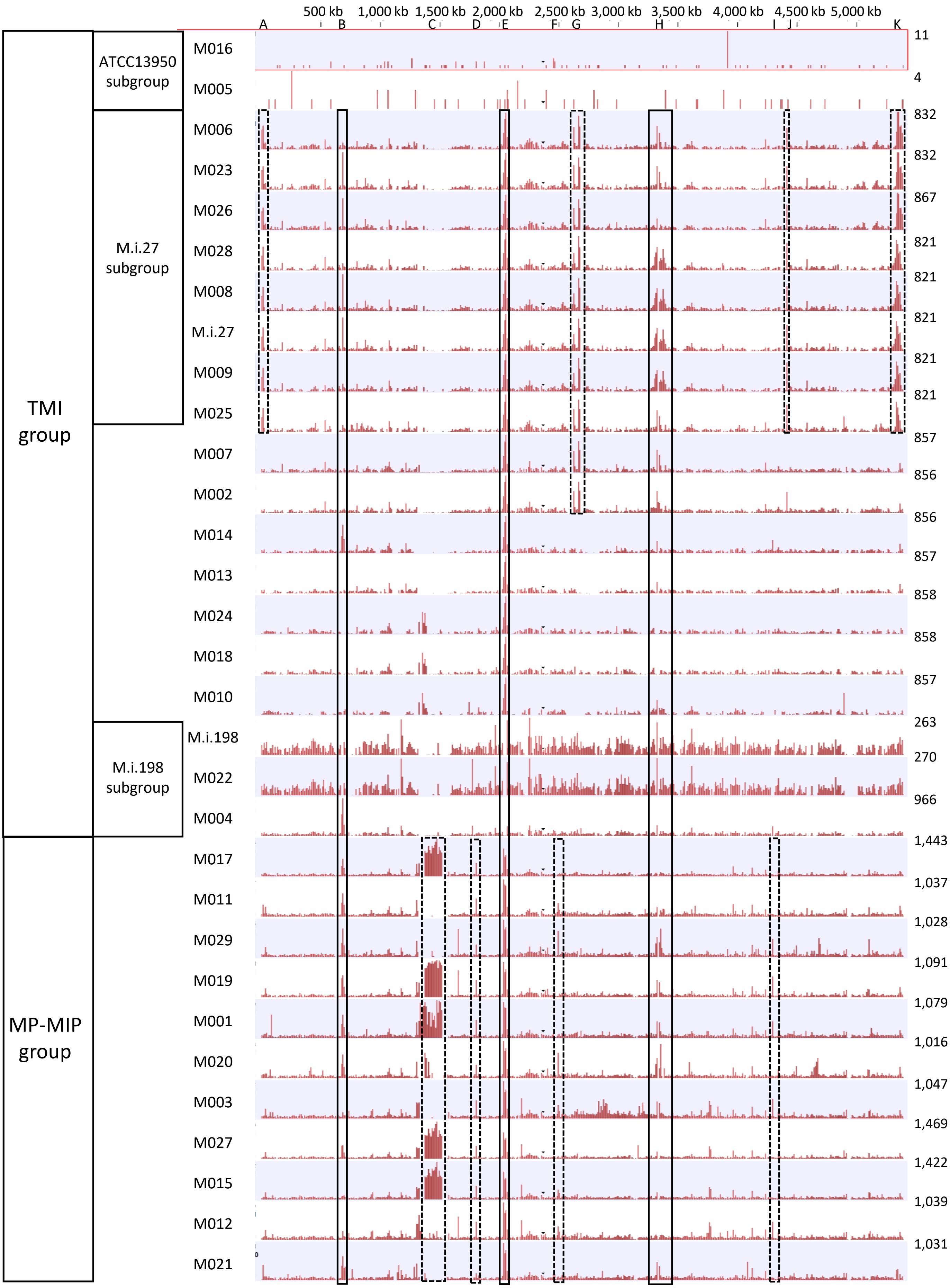

Supplemental Figure S7. Distribution of single nucleotide variants (SNV) throughout the genome.

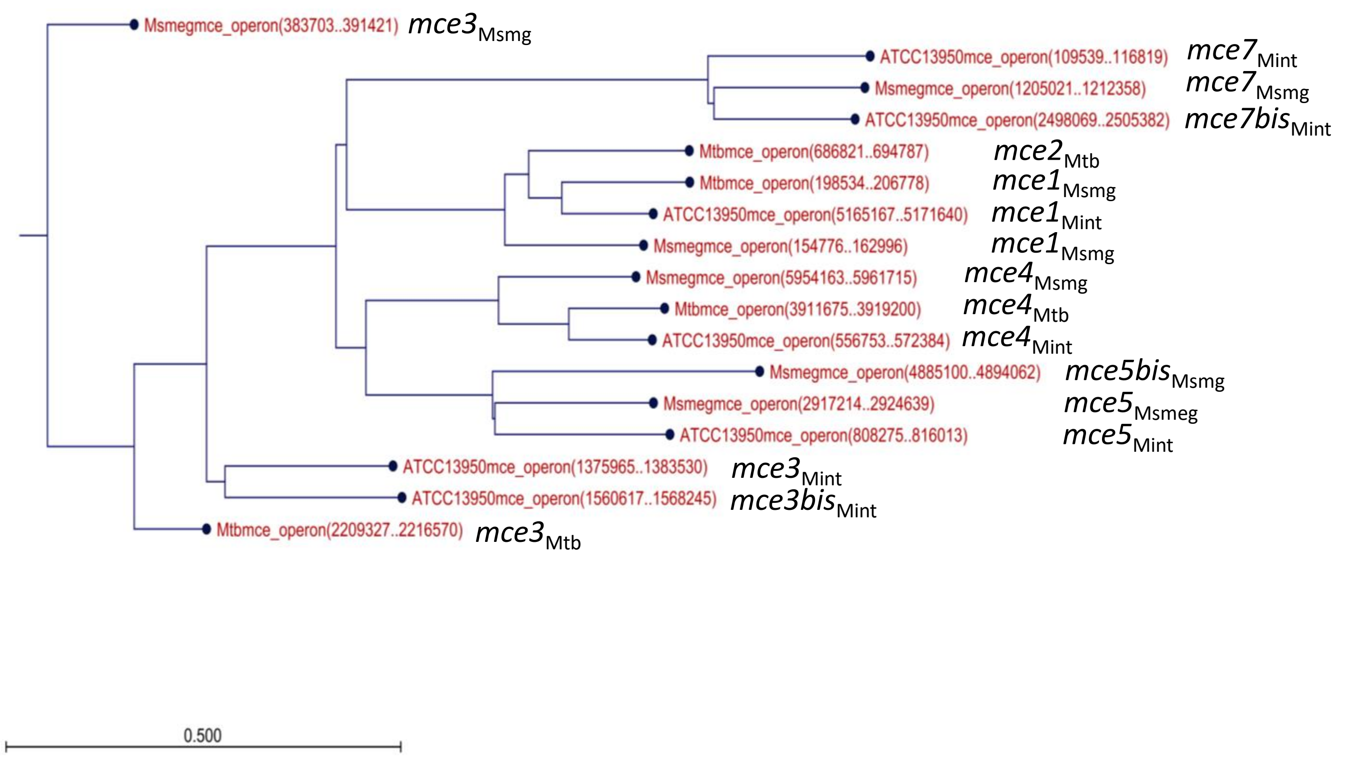

Supplemental Figure S8. Phylogenetic tree of *mce* operons between *M. tuberculosis* H37Rv, *M. smegmatis* mc^{2}155 and *M. intracellulare* ATCC13950. The tree was generated by using the Neighbor-Joining method.

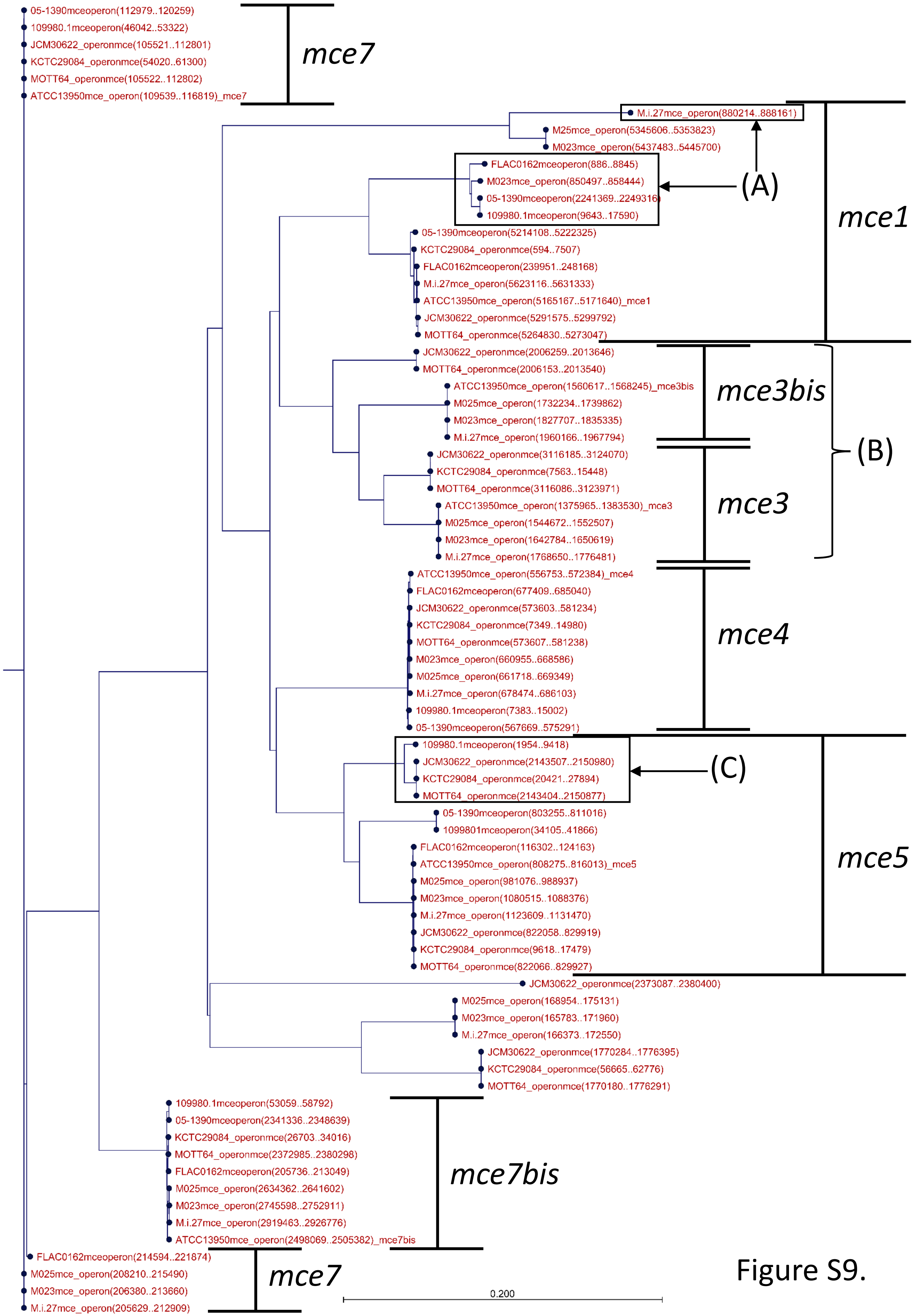
05-1390mceoperon(112979..120259)
109980.1mceoperon(46042..53322)
JCM30622_operonmce(105521..112801)
KCTC29084_operonmce(54020..61300)
MOTT64_operonmce(105522..112802)
ATCC13950mce_operon(109539..116819)_mce7
mce7
M.i.27mce_operon(880214..888161)
M25mce_operon(5345606..5353823)
M023mce_operon(5437483..5445700)
FLAC0162mceoperon(886..8845)
M023mce_operon(850497..858444)
05-1390mceoperon(2241369..2249316)
109980.1mceoperon(9643..17590)
(A)
05-1390mceoperon(5214108..5222325)
KCTC29084_operonmce(594..7507)
FLAC0162mceoperon(239951..248168)
M.i.27mce_operon(5623116..5631333)
ATCC13950mce_operon(5165167..5171640)_mce1
JCM30622_operonmce(5291575..5299792)
MOTT64_operonmce(5264830..5273047)
mce1
JCM30622_operonmce(2006259..2013646)
MOTT64_operonmce(2006153..2013540)
ATCC13950mce_operon(1560617..1568245)_mce3bis
M025mce_operon(1732234..1739862)
M023mce_operon(1827707..1835335)
M.i.27mce_operon(1960166..1967794)
mce3bis
JCM30622_operonmce(3116185..3124070)
KCTC29084_operonmce(7563..15448)
MOTT64_operonmce(3116086..3123971)
ATCC13950mce_operon(1375965..1383530)_mce3
M025mce_operon(1544672..1552507)
M023mce_operon(1642784..1650619)
M.i.27mce_operon(1768650..1776481)
mce3
(B)
ATCC13950mce_operon(556753..572384)_mce4
FLAC0162mceoperon(677409..685040)
JCM30622_operonmce(573603..581234)
KCTC29084_operonmce(7349..14980)
MOTT64_operonmce(573607..581238)
M023mce_operon(660955..668586)
M025mce_operon(661718..669349)
M.i.27mce_operon(678474..686103)
109980.1mceoperon(7383..15002)
05-1390mceoperon(567669..575291)
mce4
109980.1mceoperon(1954..9418)
JCM30622_operonmce(2143507..2150980)
KCTC29084_operonmce(20421..27894)
MOTT64_operonmce(2143404..2150877)
(C)
05-1390mceoperon(803255..811016)
1099801mceoperon(34105..41866)
FLAC0162mceoperon(116302..124163)
ATCC13950mce_operon(808275..816013)_mce5
M025mce_operon(981076..988937)
M023mce_operon(1080515..1088376)
M.i.27mce_operon(1123609..1131470)
JCM30622_operonmce(822058..829919)
KCTC29084_operonmce(9618..17479)
MOTT64_operonmce(822066..829927)
mce5
JCM30622_operonmce(2373087..2380400)
M025mce_operon(168954..175131)
M023mce_operon(165783..171960)
M.i.27mce_operon(166373..172550)
JCM30622_operonmce(1770284..1776395)
KCTC29084_operonmce(56665..62776)
MOTT64_operonmce(1770180..1776291)
109980.1mceoperon(53059..58792)
05-1390mceoperon(2341336..2348639)
KCTC29084_operonmce(26703..34016)
MOTT64_operonmce(2372985..2380298)
FLAC0162mceoperon(205736..213049)
M025mce_operon(2634362..2641602)
M023mce_operon(2745598..2752911)
M.i.27mce_operon(2919463..2926776)
ATCC13950mce_operon(2498069..2505382)_mce7bis
mce7bis
FLAC0162mceoperon(214594..221874)
M025mce_operon(208210..215490)
M023mce_operon(206380..213660)
M.i.27mce_operon(205629..212909)
mce7
0.200

Figure S9.

Supplemental Figure S9. Phylogenetic tree of *mce* operons among the strains of the TMI group (M.i.27, M023, M025, FLAC0162, ATCC13950), the MP-MIP group (MOTT64, KCTKC29084, JCM30622) and *M. yongonense* (109980.1, 05-1390). (A) There was an additional *mce1* operon in the strains of the TMI group and *M. yongonense* not found in those of the MP-MIP group. (B) *Mce3* operons (*mce3* and *mce3bis*) were present only in the strains of the groups of TMI and MP-MIP not found in those of *M. yongonense*. (C) There was an additional *mce5* operon in the strains of the MP-MIP group and *M. yongonense* strain 109980.1 not found in those of the TMI group.

Figure S10
(A)

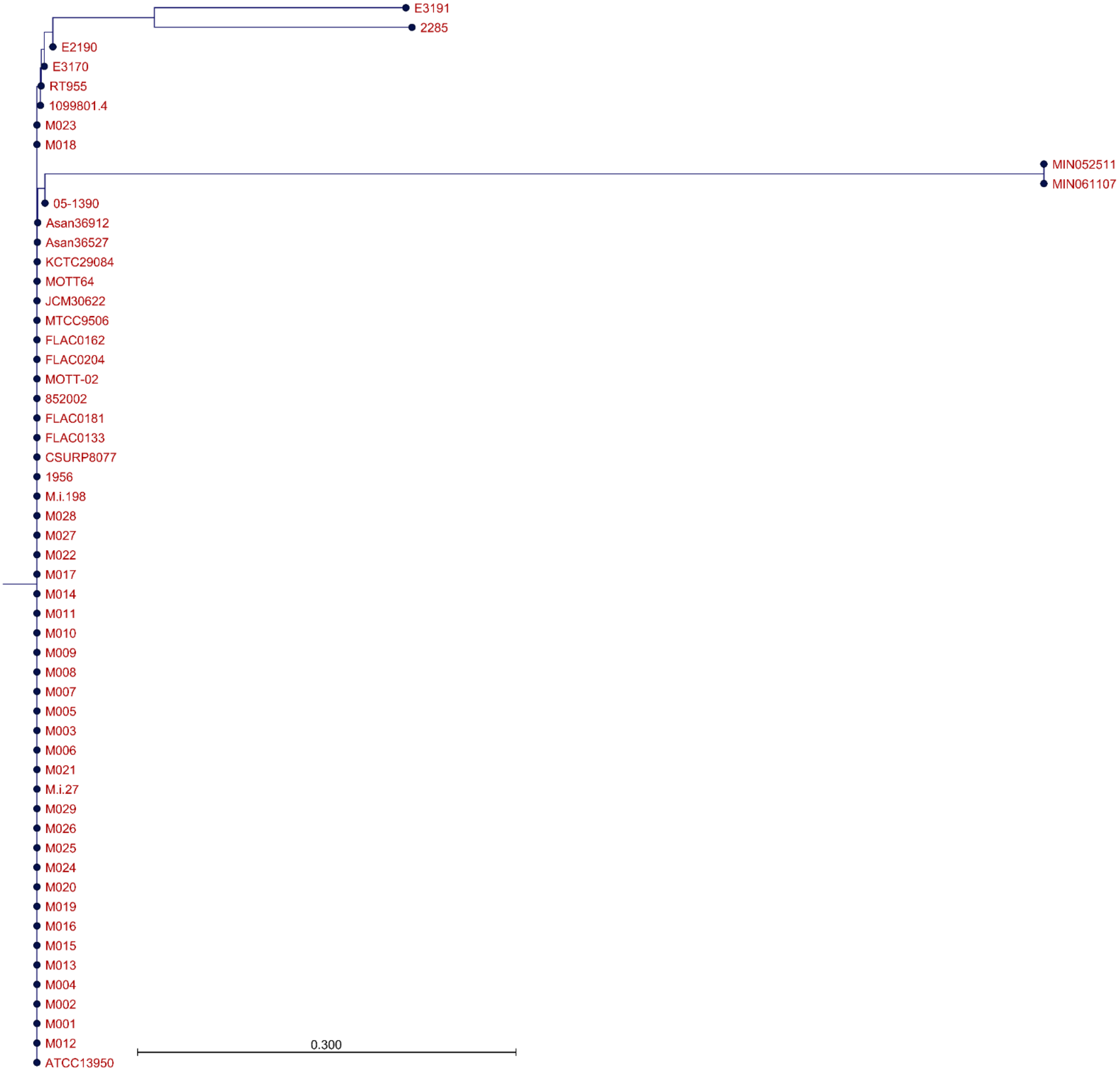

Supplemental Figure S10. Phylogenic trees based on the sequences of the housekeeping genes. The sequences extracted from the whole genome sequencing data based on the Ref 18 (Kim S-Y et al. BMC Infect Dis. 2015) were aligned using the analytic tool provided by the CLC Genomics Workbench system. (A) Phylogenic tree based on the 16S-23S ribosomal internal transcribed space (ITS) sequence. (B) Phylogenic tree based on the *hsp65* sequence. (C) Phylogenic tree based on the *rpoB* (Region V) sequence. (D) Phylogenic tree based on the *hsp65-rpoB* concatenated sequence. (E) Phylogenic tree based on the *hsp65-rpoB*-ITS concatenated sequence.

Figure S10 Continued
(B)

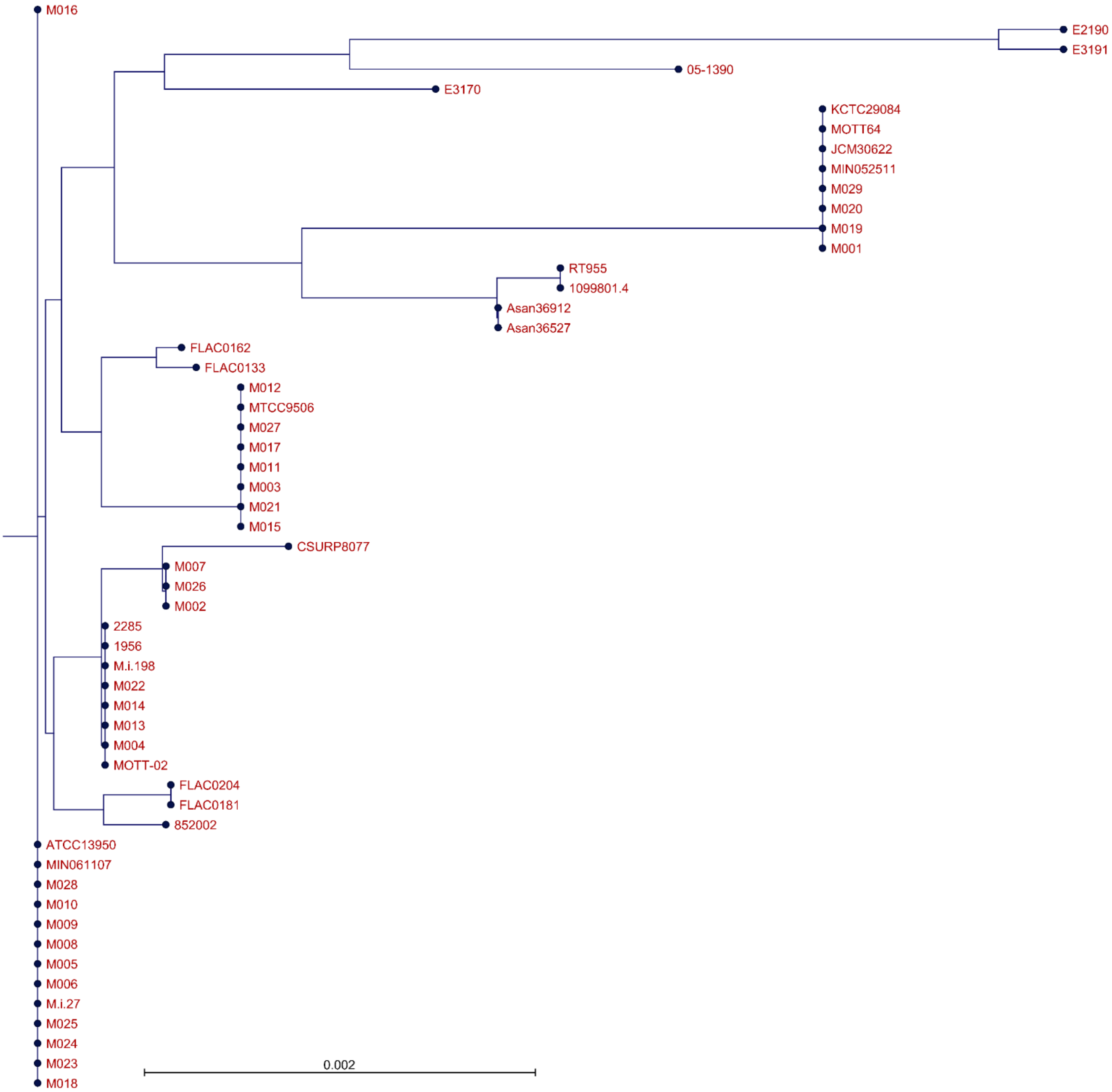

Figure S10 (Continued)
(C)

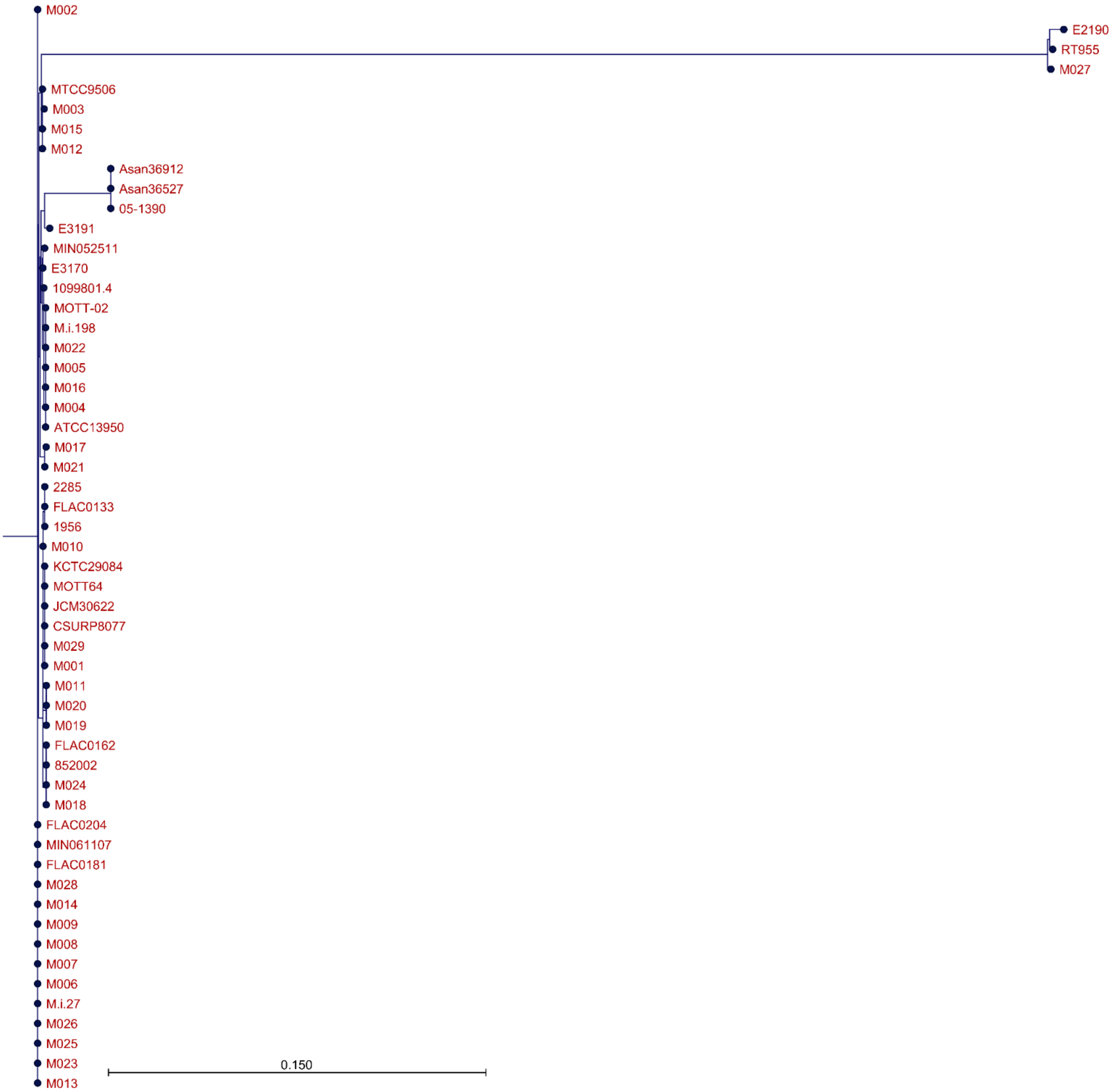

Figure S10 (Continued)
(D)

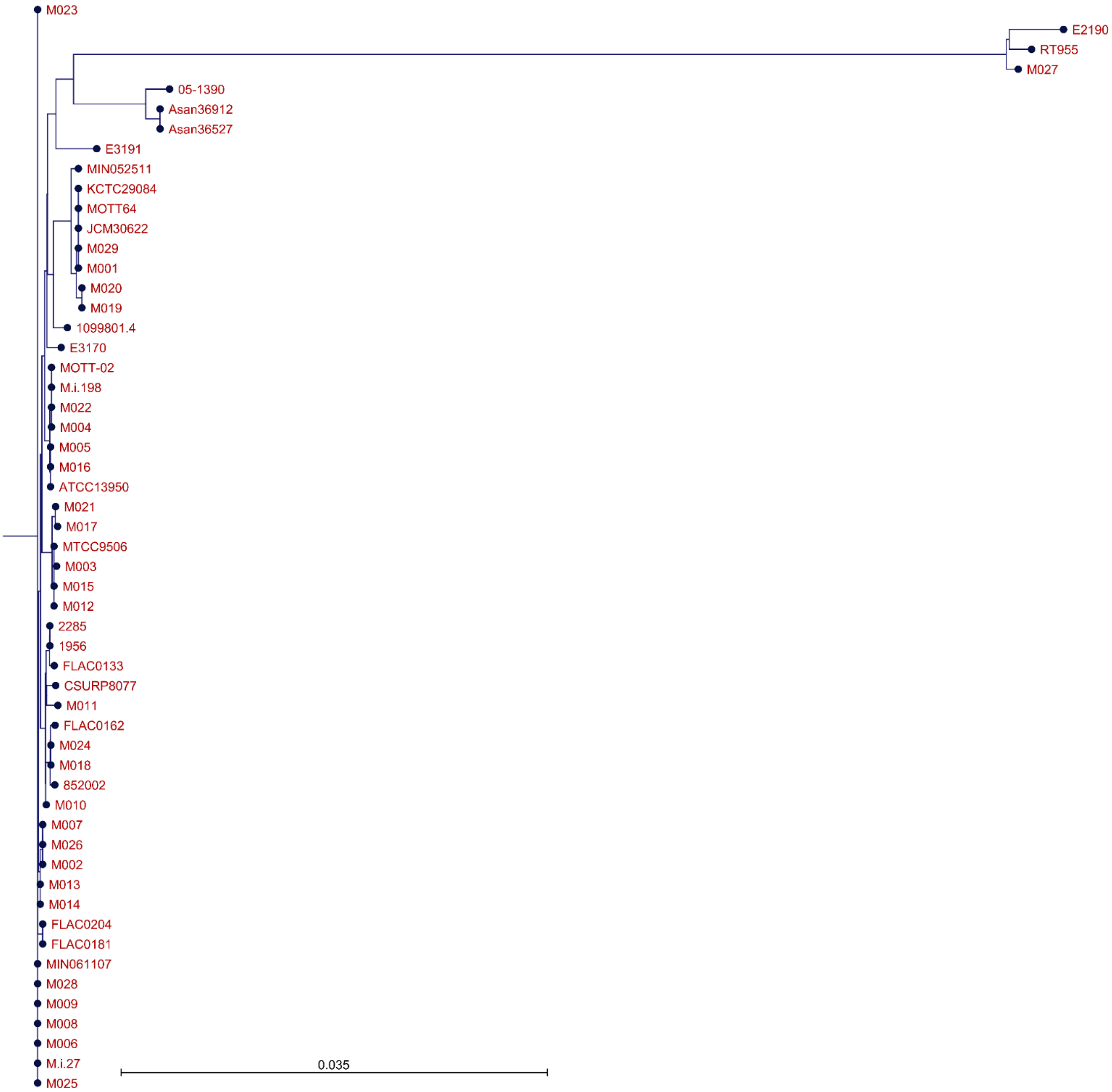

Figure S10 (Continued)
(E)

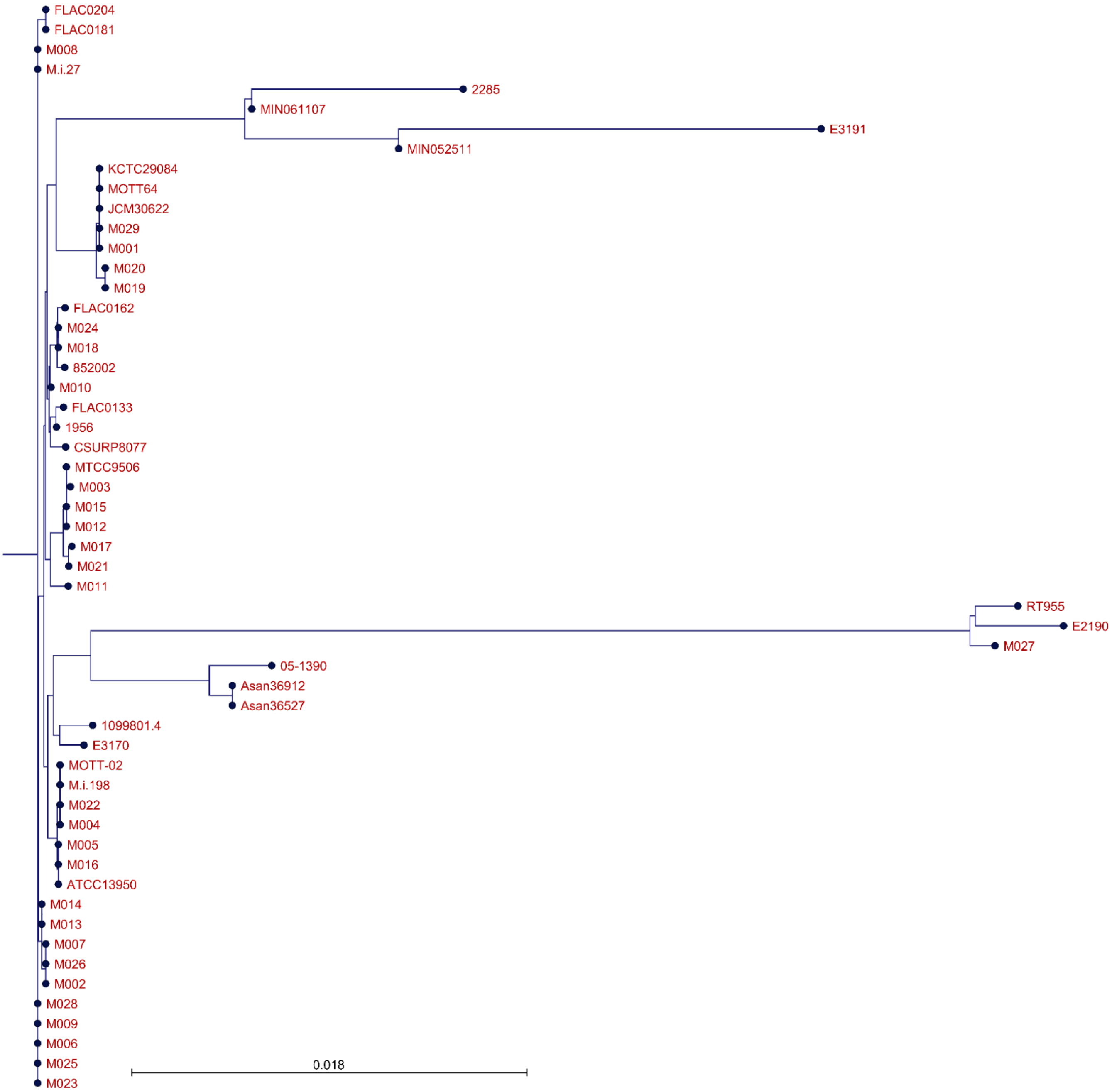

Supplement: Supplementary file 1 — Additional file 1. [file 12866_2021_2163_MOESM1_ESM.zip › 20210217Figures_supplementaryexceptFigureS3.pdf]
